# Competitiveness for Nodule Colonization in Sinorhizobium meliloti: Combined *In Vitro*-Tagged Strain Competition and Genome-Wide Association Analysis

**DOI:** 10.1128/mSystems.00550-21

**Published:** 2021-07-27

**Authors:** Agnese Bellabarba, Giovanni Bacci, Francesca Decorosi, Erki Aun, Elisa Azzarello, Maido Remm, Luciana Giovannetti, Carlo Viti, Alessio Mengoni, Francesco Pini

**Affiliations:** a Department of Agronomy, Food, Environmental and Forestry (DAGRI), University of Florence, Sesto Fiorentino, Italy; b Genexpress Laboratory, Department of Agronomy, Food, Environmental and Forestry (DAGRI), University of Florence, Sesto Fiorentino, Italy; c Department of Biology, University of Florence, Sesto Fiorentino, Italy; d Department of Bioinformatics, Institute of Molecular and Cell Biology, University of Tartu, Tartu, Estonia; e Department of Biology, University of Bari Aldo Moro, Bari, Italy; Wageningen University

**Keywords:** GWAS, competition, *Sinorhizobium meliloti*, rhizobia, legume

## Abstract

Associations between leguminous plants and symbiotic nitrogen-fixing rhizobia are a classic example of mutualism between a eukaryotic host and a specific group of prokaryotic microbes. Although this symbiosis is in part species specific, different rhizobial strains may colonize the same nodule. Some rhizobial strains are commonly known as better competitors than others, but detailed analyses that aim to predict rhizobial competitive abilities based on genomes are still scarce. Here, we performed a bacterial genome-wide association (GWAS) analysis to define the genomic determinants related to the competitive capabilities in the model rhizobial species Sinorhizobium meliloti. For this, 13 tester strains were green fluorescent protein (GFP) tagged and assayed versus 3 red fluorescent protein (RFP)-tagged reference competitor strains (Rm1021, AK83, and BL225C) in a Medicago sativa nodule occupancy test. Competition data and strain genomic sequences were employed to build a model for GWAS based on *k*-mers. Among the *k*-mers with the highest scores, 51 *k*-mers mapped on the genomes of four strains showing the highest competition phenotypes (>60% single strain nodule occupancy; GR4, KH35c, KH46, and SM11) versus BL225C. These *k*-mers were mainly located on the symbiosis-related megaplasmid pSymA, specifically on genes coding for transporters, proteins involved in the biosynthesis of cofactors, and proteins related to metabolism (e.g., fatty acids). The same analysis was performed considering the sum of single and mixed nodules obtained in the competition assays versus BL225C, retrieving *k*-mers mapped on the genes previously found and on *vir* genes. Therefore, the competition abilities seem to be linked to multiple genetic determinants and comprise several cellular components.

**IMPORTANCE** Decoding the competitive pattern that occurs in the rhizosphere is challenging in the study of bacterial social interaction strategies. To date, the single-gene approach has mainly been used to uncover the bases of nodulation, but there is still a knowledge gap regarding the main features that *a priori* characterize rhizobial strains able to outcompete indigenous rhizobia. Therefore, tracking down which traits make different rhizobial strains able to win the competition for plant infection over other indigenous rhizobia will improve the strain selection process and, consequently, plant yield in sustainable agricultural production systems. We proved that a *k*-mer-based GWAS approach can efficiently identify the competition determinants of a panel of strains previously analyzed for their plant tissue occupancy using double fluorescent labeling. The reported strategy will be useful for detailed studies on the genomic aspects of the evolution of bacterial symbiosis and for an extensive evaluation of rhizobial inoculants.

## INTRODUCTION

The nitrogen-fixing symbiotic interaction between rhizobia and legumes (mostly Fabaceae) is a classic example of a mutualistic association (1). It starts with the mutual recognition of molecular signals, specifically flavonoids released from the plant roots and Nod factors produced by rhizobia (2). Nod factors induce a molecular response in plant root cells, which ultimately leads to rhizobium entry in the radical tissue and intracellular colonization (3). Molecular signaling also drives the development of new structures on plant roots, called nodules, where intracellular rhizobia differentiate into bacteroids, the form responsible for dinitrogen fixation (4–6). In a single nodule (a mass of a few hundreds of milligrams), up to 10^6^ bacterial cells can be recovered, whereas in the soil, free-living rhizobia do generally not exceed 10^3^ to 10^4^/g of soil (7, 8).

As in a trade framework, the benefit for the rhizobium is obtaining a protected environment where it can reproduce (under control) and receive carbon and energy supplies from the plant, whereas the reward for the plant is the availability of fixed nitrogen (7, 9). Since rhizobial transmission is horizontal, plants may also be colonized by poorly effective (low-nitrogen-fixing) strains. However, host plants could control the colonization by inefficient strains via sanctioning root nodules and limiting their growth (10–13). Moreover, the presence of multiple strains within a nodule (mixed nodule) may occur (14, 15). Under these circumstances, inefficient rhizobia can behave as cheaters, decreasing the overall nitrogen-fixing performance (7, 14). Consequently, understanding the mechanisms underlying strain competition has great importance for fully understanding the evolution of symbiosis (16, 17) and predicting rhizobial inoculant efficiency (18). The genetic bases of competitiveness among rhizobial strains are still elusive. To date, most of the studies have identified symbiotic genetic determinants from experiments carried out with mutants of a few different natural strains (7).

The link between a phenotype and its genetic basis, hence predicting phenotypes from the sole genomic information, is one of the challenges of biology (19). Genome-wide association studies (GWASs) are commonly used for identifying the putative functional role of a set of allelic variations in groups of individuals. In bacteria, GWASs have been applied to several species for predicting complex (i.e., multigenic) phenotypes, such as antibiotic tolerance and host interaction (20–22). However, most of the studies investigated phenotypes under strong selective pressure, whereas it is still challenging to determine the genetic basis of phenotypes under mild selection (23). The identification of the genetic determinants in host-bacterium interactions is essential for the improvement of sustainable agriculture: GWASs on plant holobionts (the ensemble of the plant and the other organisms living in or around it) have been proposed (73–74), aiming to provide the basis for future breeding programs, which includes, among the plant traits, the recruitment of the “good” microbiome.

Recent studies have reported the feasibility of experimental setups combining symbiotic assays with genome sequencing approaches and GWASs in rhizobia to define the genetic determinants of symbiotic performances (24–27). In the model rhizobium Sinorhizobium meliloti, association analyses have been employed to explore the genetic basis of various phenotypic traits, including antibiotic resistance and symbiotic and metabolic traits (24). A select-and-resequence approach has successfully been applied to measure the fitness of a set of 101 S. meliloti strains with two genotypes of the host plant Medicago truncatula (25). However, the predictive value of single rhizobial genotypes (i.e., genomes) toward the expected fitness in terms of competitive capabilities to establish a successful symbiosis is still unclear. To date, although many genetic details of the symbiotic interaction are known for single strain colonization (28), we still do not know which rhizobial features increase the chances to win the competition for plant infection, outcompeting other indigenous rhizobia. Unearthing these genetic determinants can advance our knowledge on the genomic aspects of the evolution of bacterial symbiosis and may have a direct application in the screening and amelioration of rhizobial inoculants for sustainable agricultural production systems.

The main objective of this work was to identify a set of candidate genes which may increase rhizobial competition capabilities. We chose as model rhizobial species *Ensifer* (syn. *Sinorhizobium*) *meliloti*; abundant molecular genetics data and tools are available for this bacterium (29), a good number of strains was sequenced, and preliminary data on symbiotic performances and competition are available (14, 20, 21, 25, 30). The competition phenotype was measured by performing a series of nodulation assays where pairs of fluorescently labeled S. meliloti strains were used to infect alfalfa (Medicago sativa) plants. The obtained data were then coupled with the genomic sequences of the same strains to perform a genome-wide association analysis.

## RESULTS

### Construction of fluorescently tagged S. meliloti strains.

To set up *in vitro* tests for measuring competition capabilities, a panel of 16 S. meliloti strains was selected. Three well-characterized strains for competition capabilities (S. meliloti BL225C, AK83, and Rm1021) were chosen (14) and used as reference competitors versus 13 S. meliloti strains (tester strains) whose genome sequences were available (see Table S1 in the supplemental material). The phylogenetic relationships among the 13 S. meliloti strains were evaluated (Fig. S1A), and their pangenome was analyzed (Fig. S1B to D). The pangenome was composed of 15,419 genes: 4,278 were shared by all strains (core genome) and 6,622 were strain specific (Fig. S1D). For all above-mentioned 13 tester strains, green fluorescent protein (GFP) derivatives were constructed by cloning the pHC60 plasmid, which constitutively expresses the GFP. Strains S. meliloti Rm1021, BL225C, and AK83 were tagged with red fluorescent protein (RFP) by using the pBHR mRFP plasmid. Preliminary single inoculation assays were performed, showing that all strains were able to form nodules on the roots of alfalfa plants (Fig. S2). For all but two strains (M270 and T073), nitrogenase activity inside the nodules was detected (Fig. S2D), in agreement with previous results that showed low nitrogen fixation abilities in symbiotic interaction with *M. truncatula* (31). Differences in nodulation, plant growth, and nitrogenase activity among strains were observed. Strains S. meliloti AK58, RU11/001, SM11, USDA1157, GR4, and CCMM B554 showed the highest values of nitrogenase activity and plant growth promotion (Fig. S2).

10.1128/mSystems.00550-21.2 FIG S1 Evolutionary relationships and pangenomes of strains used as competitors. (A) The evolutionary history was inferred using the UPGMA method (unweighted pair group method using average linkages) on core gene concatemer alignment. The optimal tree with the sum of branch length = 0.02977589 is shown. The percentage of replicate trees in which the associated taxa clustered together in the bootstrap test (10,000 replicates) is shown next to the branches. The evolutionary distances were computed using the maximum composite likelihood method and are provided in the units of the number of base substitutions per site. We obtained a total of 3,998,704 positions in the final data set. (B) Heatmap showing gene presence (dark blue) or absence (light blue) in each strain; the tree on the left was built based on presence/absence of genes. (C) Histogram showing the frequency of genes depending on the number of genomes. (D) Pie chart displaying all genes present in the pangenome and their breakdown in the different genomes. Download FIG S1, PDF file, 1.4 MB. Copyright © 2021 Bellabarba et al. 2021 Bellabarba et al. https://creativecommons.org/licenses/by/4.0/ This content is distributed under the terms of the Creative Commons Attribution 4.0 International license.

10.1128/mSystems.00550-21.3 FIG S2 Nodulation assay and nitrogen fixation efficiency with single strains. (A) Number of nodules/plant; (B) epicotyl length; (C) plant dry weight; (D) acetylene reduction assay (ARA). Different letters indicate significant differences between treatments (*P* value < 0.05). Download FIG S2, TIF file, 2.7 MB. Copyright © 2021 Bellabarba et al. 2021 Bellabarba et al. https://creativecommons.org/licenses/by/4.0/ This content is distributed under the terms of the Creative Commons Attribution 4.0 International license.

10.1128/mSystems.00550-21.8 TABLE S1 Sinorhizobium meliloti strains used in this work. Download Table S1, DOCX file, 0.02 MB. Copyright © 2021 Bellabarba et al. 2021 Bellabarba et al. https://creativecommons.org/licenses/by/4.0/ This content is distributed under the terms of the Creative Commons Attribution 4.0 International license.

### Competition capabilities for nodule colonization differ in relation to competitor counterparts.

Tagged S. meliloti strains were used in a set of competition experiments: each with a GFP-tagged strain (13 strains in total) versus an RFP-tagged competitor strain (S. meliloti BL225C, AK83, or Rm1021; 13 × 3, total of 39). A large variability in nodule colonization was observed among and within the three sets of competition experiments (versus Rm1021, versus AK83, and versus BL225C). The three competition tests showed differences in the number of total nodules produced per plant (*P* value < 0.001, Fig. S3A); in the competition experiments with AK83, the highest number of nodules was observed (Fig. S3A).

10.1128/mSystems.00550-21.4 FIG S3 Number of nodules in the three competitions. (A) Total nodules per plant. (B) Total mixed nodules per plant. Different letters indicate significant differences between treatments (*P* value < 0.05). Download FIG S3, TIF file, 1.1 MB. Copyright © 2021 Bellabarba et al. 2021 Bellabarba et al. https://creativecommons.org/licenses/by/4.0/ This content is distributed under the terms of the Creative Commons Attribution 4.0 International license.

**FIG 1:**
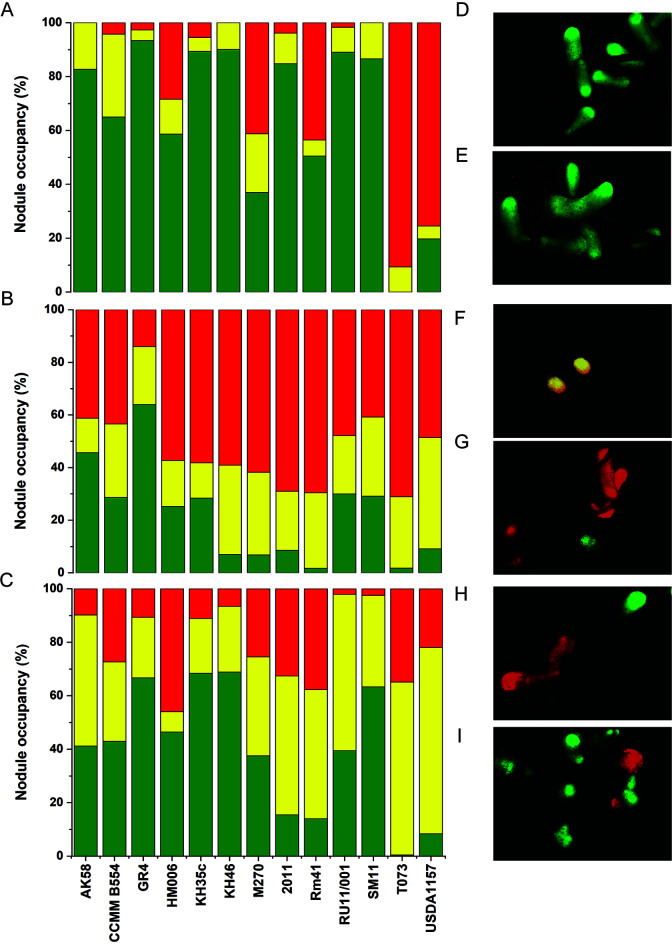
Competition performances and epifluorescence stereomicroscope images. Bar plots showing the percentages of nodule occupancy of 13 Sinorhizobium meliloti strains in three sets of competition experiments: competition versus S. meliloti Rm1021 (A), competition versus S. meliloti AK83 (B), and competition versus S. meliloti BL225C (C). Green bars represent single nodule occupancy of the strains tested whose ID is reported on the *x* axis; in yellow, the percentage of mixed nodules is shown (nodules occupied by both strains), and in red, the single nodule occupancy of the competitor used in each set of experiments is shown. Pictures show nodules of *Medicago sativa* inoculated with a mix of S. meliloti 1021 RFP-tagged and KH46 GFP-tagged (D) or GR4 GFP-tagged (E), S. meliloti AK83 RFP-tagged and HM006 GFP-tagged (F and G), and S. meliloti BL225C RFP-tagged and CCMM B554 GFP-tagged (H) or RU11/001 GFP-tagged (I).

Competition capabilities were evaluated as single nodule occupancy (nodules colonized by a single strain) of the tested strain in respect to a reference strain; good competitors were characterized by a single nodule occupancy higher than 60%, medium competitors between 20 and 60%, and weak competitors below 20%. In competition with Rm1021, most of the strains outperformed (Fig. 1A). This competition test was characterized by a high average value of nodule occupancy of the tested strains, equal to 65.12%. Most of the strains showed a single nodule occupancy higher than 60%, with the highest value (93.4%) observed for GR4. Three strains (HM006, Rm41, and M270) exhibited medium competition capabilities, and two strains (T073 and USDA1157) poorly performed (Fig. 1A and Table S2). Among the three strains with medium competition capabilities, HM006 and Rm41 displayed values close to those of high-performing strains (58.6 and 50.5%, respectively), whereas M270 nodule occupancy was 37%. Medium-low performance of strains M270 and T073 (T073 competition was characterized by the presence of mixed nodules or nodules with strain Rm1021 only) may be related to nodule sanctioning (plant limiting nutrients to inefficient nodules), as T073 and M270 were unable to fix nitrogen (Fig. S2D).

10.1128/mSystems.00550-21.9 TABLE S2 Single nodule occupancy and sum of single and mixed nodules (in brackets) of Sinorhizobium meliloti tested strains in the competition experiments versus S. meliloti strains Rm1021, AK83, and BL225C. Different letters indicate significant differences (Kruskal-Wallis and Dunn test, *P* value < 0.05) within a competition assay (columns; versus BL225C, versus AK83, and versus Rm1021). Download Table S2, DOCX file, 0.02 MB. Copyright © 2021 Bellabarba et al. 2021 Bellabarba et al. https://creativecommons.org/licenses/by/4.0/ This content is distributed under the terms of the Creative Commons Attribution 4.0 International license.

In the competition experiments with AK83, conversely to the pattern highlighted with Rm1021, a general decrease in nodule occupancy of the 13 strains tested was observed (Fig. 1B), resulting in a lower average value of nodule occupancy (21.99%; Fig. 1B and Table S2). Except for GR4, showing the highest percentage of occupancy (63.9%), all strains displayed weak-medium competitive capabilities (nodule occupancy lower than 60%; Fig. 1B and Table S2). The lowest values of nodule occupancy were detected for Rm41 and T073 (1.7 and 1.8%, respectively).

Lastly, in the competition with BL225C (Fig. 1C and Table S2), the average value of nodule occupancy of the strains tested was equal to 39.45%. The most competitive strains were GR4, KH35c, KH46, and SM11, showing nodule occupancies ranging from 63.4 to 68.9%. The lowest percentage of nodule occupancy, 0.4%, was detected for T073.

In both competition with AK83 and that with BL225C, a higher abundance of mixed nodules (nodules infected by both S. meliloti strains) was observed compared to competition with Rm1021 (Fig. S3B). Considering single nodule occupancy of the whole data set (mean values), we may roughly divide the strains into two groups: one containing highly competitive strains (formed by GR4, KH35c, AK58, KH46, SM11, RU11/001, CCMM B554, and HM006) and one with low-performance strains (composed of T073, USDA1157, Rm41, M270, and 2011) (Fig. S4A; permutational multivariate analysis of variance [PERMANOVA], Bonferroni-corrected *P* value = 0.0009). The same subdivision could be observed also considering the sum of single occupied nodules and mixed nodules, with the only exception of strain HM006, which moved from the highly competitive group to the low one (Fig. S4B; PERMANOVA, Bonferroni-corrected *P* value = 0.0005).

10.1128/mSystems.00550-21.5 FIG S4 Principal-component analysis (PCA) based on Sinorhizobium meliloti strain nodule occupancy. (A) Single-occupied nodules. (B) Single- and mixed-occupied nodules. Download FIG S4, TIF file, 1.0 MB. Copyright © 2021 Bellabarba et al. 2021 Bellabarba et al. https://creativecommons.org/licenses/by/4.0/ This content is distributed under the terms of the Creative Commons Attribution 4.0 International license.

### Putative genetic determinants associated with good competition capabilities versus S. meliloti BL225C.

Short DNA oligomers with constant length *k*, termed *k*-mers, allow capture of a large set of genetic variants in a population, including single nucleotide polymorphisms (SNPs) and insertions/deletions (indels) (20, 30). To pinpoint genetic determinants that might be responsible for the competition capability variation among S. meliloti strains, we performed an association analysis for each competition assay using PhenotypeSeeker (32) (Table 1). Nested cross-validation analyses were also performed to evaluate the competition phenotype predictabilities (see Text S1 in the supplemental material). The genome sequences of the tested strains and a matrix based on the competition phenotype, considered the single nodule occupancy (Table S2), were used to identify specific *k*-mers significantly associated with competition phenotypes (*P* value < 0.05). Due to the greater extent of positive performances, ranging from 50.5 to 93.4%, shown by most of the strains tested versus Rm1021 (Fig. 1A; Table S2), a large amount of total *k*-mers significantly associated with this competition phenotype was obtained (Table 1). In contrast, the number of total *k*-mers identified in tested strains related to the competition versus BL225C was smaller in comparison with other data sets (Table 1). Moreover, the wider *P* value range for significantly associated *k*-mers was found in the analysis of competition versus BL225C, which was also characterized by higher *P* values (Table 1). Therefore, for the subsequent steps, we selected the set of *k*-mers related to the competition versus BL225C. This set was mapped on the genomes of the 13 tested strains to retrieve the genetic determinants associated with the competition phenotype.

**TABLE 1:** Identification of significant *k*-mers by association analysis with PhenotypeSeeker^*a*^

Data set		Total *k*-mers (*P* value < 0.05)	*P* value range
vs Rm1021	Single nodule occupancy	4.39E^+05^	4.99E^−02^–4.31E^−03^
	(Single and mixed nodule occupancy)	(4.36E^+05^)	(4.88E^−02^–1.07E^−02^)

vs BL225C	Single nodule occupancy	1.82E^+05^	4.98E^−02^–1.35E^−05^
	(Single and mixed nodule occupancy)	(3.60E^+05^)	(4.96E^−02^–5.14E^−05^)

vs AK83	Single nodule occupancy	2.92E^+05^	4.97E^−02^–1.05E^−03^
	(Single and mixed nodule occupancy)	(1.70E^+05^)	(4.99E^−02^–6.15E^−04^)

a The numbers of total *k*-mers associated with the competition phenotype (*P* value < 0.05) for the three competition experiments and their range of *P* values are reported.

10.1128/mSystems.00550-21.1 TEXT S1 Supplementary results and discussion: putative genetic determinants associated with increased competition and coinfecting nodule capabilities in assays versus Sinorhizobium meliloti BL225C (functions detected in single strains only). Modeling competition pattern from genome sequences. Supplementary methods: nodulation and acetylene reduction assays. Annotation and phylogenetic analyses. Mapping procedure. Download Text S1, DOCX file, 0.03 MB. Copyright © 2021 Bellabarba et al. 2021 Bellabarba et al. https://creativecommons.org/licenses/by/4.0/ This content is distributed under the terms of the Creative Commons Attribution 4.0 International license.

**FIG 2:**
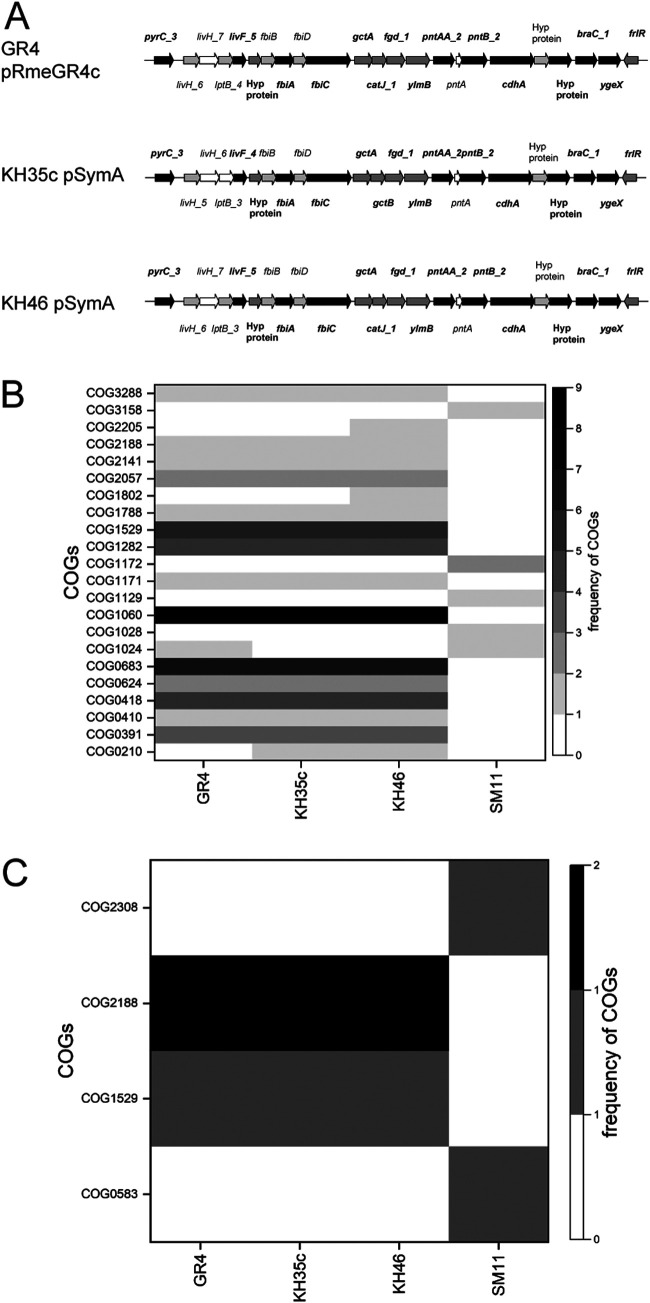
Genetic determinants associated with single nodule occupancy. (A) *k*-mers mapping in a region of the symbiotic megaplasmid (pSymA or homolog plasmids) present exclusively in the genomes of Sinorhizobium meliloti GR4, KH35c, and KH46. Genes containing one or more *k*-mers are indicated with a colored arrow (black or shades of gray): black arrows indicate genes retrieved in both single and single plus mixed nodule occupancy data sets, dark gray arrows indicate genes retrieved in the single nodule occupancy data set only, and light gray arrows indicate genes retrieved in the single plus mixed nodule occupancy data set only; gene annotation is referred to the Prokka output. (B and C) Frequency of candidate functions of gene hits (B) and regulatory regions (C) identified by 51 best *k*-mers in the most competitive strains. The frequency of candidate functions reported as COG annotations (rows) in each strain (columns) is represented by grayscale shades.

Among the top *k*-mers (*P* value threshold < 0.001, see supplemental File S2A at https://doi.org/10.5061/dryad.x95x69pj5), 51 *k*-mers (*P* value = 1.31 × 10^−4^) mapped in genomes of the four strains that showed single nodule occupancy higher than 60% (S. meliloti GR4, SM11, KH35c, and KH46) in the competition test versus BL225C (see supplemental File S1 at https://doi.org/10.5061/dryad.x95x69pj5, highlighted in bold). These best *k*-mers tagged 103 predicted protein-coding sequences (CDSs); one *k*-mer may tag multiple genes (see supplemental File S2A at https://doi.org/10.5061/dryad.x95x69pj5). Among the 103 CDSs, a set of orthologous genes was identified (see supplemental File S2A at https://doi.org/10.5061/dryad.x95x69pj5). These orthologous gene hits were mostly tracked in S. meliloti GR4, KH35c, and KH46 genomes (Fig. S5A) and were predominantly located on the symbiosis-required megaplasmid pSymA (ranging from 93.3% to 100%; Fig. S5B, C, and D), particularly in a specific region of 26 kb, present in the genome of these three strains only (Fig. 2A). In contrast, 60% of the orthologous gene hits in the SM11 genome were located on the chromid pSymB (Fig. S5E).

10.1128/mSystems.00550-21.6 FIG S5 Distribution of 51 best *k*-mers (single nodule occupancy) in Sinorhizobium meliloti GR4, KH35c, KH46, and SM11. (A to E) Distribution of orthologous gene hits among four S. meliloti strains (A) and distribution of orthologous genes among replicons of S. meliloti strains GR4 (B), KH35c (C), KH46 (D), and SM11 (E). (F to J) Distribution of unannotated CDSs among four S. meliloti strains (F) and distribution of unannotated CDSs among replicons of S. meliloti strains GR4 (G), KH35c (H), KH46 (I), and SM11 (J). (K to O) Distribution of putative regulatory region hits of orthologous gene hits among four S. meliloti strains (K) and among replicons of S. meliloti strains GR4 (L), KH35c (M), KH46 (N), and SM11 (O). (P to R) Distribution of regulatory region hits of unannotated CDSs among four S. meliloti strains (P) and among replicons of S. meliloti strains GR4 (Q) and SM11 (R). Download FIG S5, TIF file, 1.5 MB. Copyright © 2021 Bellabarba et al. 2021 Bellabarba et al. https://creativecommons.org/licenses/by/4.0/ This content is distributed under the terms of the Creative Commons Attribution 4.0 International license.

The distribution of the candidate function of gene hits within the most competitive strain genomes was not uniform. Enrichment for COG categories E (amino acid transport and metabolism), C (energy production and conversion), H (coenzyme transport and metabolism), and I (lipid transport and metabolism) was found in S. meliloti GR4, KH35c, and KH46. The most represented orthologous gene groups were related to the coenzyme F_420_ biosynthesis process, transmembrane transport via ABC-type systems for branched-chain amino acids, and pyrimidine nucleotide biosynthetic processes (Fig. 2B). Further, a putative caffeine dehydrogenase engaged in the pathway of caffeine transformation via C-8 oxidation and for the two subunits (PntA and PntB) of a presumptive proton-translocating NAD(P) transhydrogenase liable for NADPH balancing mechanisms (Table 2) was found. Other presumed functions common to the three strains (GR4, KH35c, and KH46) were related to amino acid degradation, carbohydrate metabolism, and oxidation-reduction, as well as transcriptional regulation by a GntR-type regulator (Table 2). The number of orthologous gene groups with functional annotation tagged by the 51 best *k*-mers was lower in SM11. Except for the orthologous group related to the fatty acid metabolic process, the candidate functions of gene hits identified in SM11 were exclusive (Table 2).

**TABLE 2:** List of functions putatively involved in promoting competing abilities^*a*^

COG ID	COG class(es)	COG functional category	Prokka annotation/other annotation	Biological process(es)
COG0210	L	Superfamily I DNA or RNA helicase	ATP-dependent DNA helicase PcrA	Mismatch repair, nucleotide excision repair
COG0391	GH	Archaeal 2-phospho-l-lactate transferase/bacterial gluconeogenesis factor, CofD/UPF0052 family	Putative phosphoenolpyruvate transferase FbiA	Coenzyme F_420_ biosynthesis
COG0410	E	ABC-type branched-chain amino acid transport system, ATPase component	High-affinity branched-chain amino acid transport ATP-binding protein LivF	High-affinity branched-chain amino acid transport
COG0418	F	Dihydroorotase	Dihydroorotase PyrC	Pyrimidine nucleotide biosynthesis
COG0624	E	Acetylornithine deacetylase/succinyl-diaminopimelate desuccinylase or related deacylase	Probable *N*-formyl-4-amino-5-aminomethyl-2-methylpyrimidine deformylase	Unknown function
COG0683	E	ABC-type branched-chain amino acid transport system, periplasmic component	Leucine-, isoleucine-, valine-, threonine-, and alanine-binding protein Brac	Branched-chain amino acid transport
COG1024	I	Enoyl-CoA hydratase/carnitine racemase	Putative fatty acid oxidation complex subunit alpha–enoyl-CoA hydratase FadJ	Fatty acid metabolism
COG1028	IQR	NAD(P)-dependent dehydrogenase, short-chain alcohol dehydrogenase family	Putative NAD-dependent glycerol dehydrogenase	Glycerol metabolic process
COG1060	H	2-Iminoacetate synthase ThiH (tyrosine cleavage enzyme, thiamine biosynthesis)	FbiC F_0_ synthase	Coenzyme F_0_ biosynthesis
COG1129	G	ABC-type sugar transport system, ATPase component	Ribose import ATP-binding protein RbsA, CUT2 family	Ribose transmembrane transport
COG1171	E	Threonine dehydratase	Diaminopropionate ammonia-lyase	Cellular amino acid catabolic process
COG1172	G	Ribose/xylose/arabinose/galactoside ABC-type transport system, permease component	Autoinducer 2 import system permease protein LsrD	AI-2 transport system
COG1282	C	NAD/NADP transhydrogenase beta subunit	NAD(P) transhydrogenase subunit beta PntB	Oxidation-reduction process; nicotinate and nicotinamide metabolism
COG1529	C	CO or xanthine dehydrogenase, Mo-binding subunit	Putative caffeine dehydrogenase subunit alpha	Oxidation-reduction process
COG1788	I	Acyl-CoA:acetate/3-ketoacid-CoA transferase, alpha subunit	Glutaconate CoA-transferase GctA subunit A	Glutamate catabolic process (via hydroxyglutarate)
COG1802	K	DNA-binding transcriptional regulator, GntR family	HTH-type transcriptional repressor RspR	DNA-binding transcriptional regulation
COG2057	I	Acyl-CoA:acetate/3-ketoacid-CoA transferase, beta subunit	Glutaconate CoA-transferase GctB subunit B	Glutamate catabolic process (via hydroxyglutarate)
COG2141	HR	Flavin-dependent oxidoreductase, luciferase family (includes alkanesulfonate monooxygenase SsuD and methylene tetrahydromethanopterin reductase)	F_420_-dependent glucose-6-phosphate dehydrogenase	Carbohydrate metabolic process, oxidation-reduction process
COG2188	K	DNA-binding transcriptional regulator, GntR family	Putative transcriptional repressor	DNA-binding transcriptional regulation
COG2205	T	K^+^-sensing histidine kinase KdpD	Two-component system sensor histidine kinase KdpD	Two-component regulatory system K^+^ sensing that regulates *kdpABCF* operon for potassium transport
COG3158	P	K^+^ transporter	Low affinity potassium transport system protein Kup	Potassium ion transport
COG3288	C	NAD/NADP transhydrogenase alpha subunit	NAD(P) transhydrogenase subunit alpha PntA	Oxidation-reduction process; nicotinate and nicotinamide metabolism

a COG description of gene hits identified by 51 *k*-mers (*P* value 1.13 × 10^−4^) in the most competitive strains (Sinorhizobium meliloti GR4, KH35c, KH46, SM11). Function/annotation are reported according to the annotation performed with Prokka in this work and/or using original annotation.

Among the 103 CDSs tagged by the 51 best *k*-mers, predicted protein-coding sequences (CDSs) with no assigned function were also identified (see supplemental File S2A at https://doi.org/10.5061/dryad.x95x69pj5). A large part of these tagged CDSs could be identified in the SM11 genome and was almost entirely located on the SM11 chromosome (Fig. S5F and J). In contrast, CDSs that were found in GR4, KH35c, and KH46 genomes were located on homologs of the symbiosis-required megaplasmid pSymA (Fig. S5G, H, and I).

Regulatory regions were also analyzed, and we considered bona fide promoter sequence hit mapping within 600 nucleotides upstream of the CDS start (30). Ten of the 51 best *k*-mers analyzed pinpointed 15 regulatory regions (see supplemental File S3A at https://doi.org/10.5061/dryad.x95x69pj5). Seven regulatory region hits were associated with CDSs with no assigned function (see supplemental File S3A at https://doi.org/10.5061/dryad.x95x69pj5). These regulatory region hits were tracked exclusively in GR4 and SM11 genomes (Fig. S5P) and were mainly located on the chromosomes of the two strains (Fig. S6Q and R). Eight regulatory region hits of putatively orthologous gene targets were identified (see Fig. S5C and see supplemental File S3A at https://doi.org/10.5061/dryad.x95x69pj5) and were entirely located on symbiosis-required megaplasmid pSymA in S. meliloti GR4, KH35c, and KH46 (Fig. S5L, M, and N) and on pSymB of S. meliloti SM11 (Fig. S5O). In GR4, KH35c, and KH46, the regulatory region hits were associated with genes encoding proteins whose functions (COG1529 and COG2188) have previously been observed (Fig. 2B and C and Table 3). In SM11, the regulatory region hits were associated with a LysR-type orthologous gene and with a gene belonging to COG2308, possibly involved in the biosynthesis of small peptides (Table 3).

**TABLE 3:** List of regulatory regions putatively involved in promoting competing abilities^*a*^

COG ID	COG class	COG functional category	Prokka annotation/product	Biological process(es)
COG2308	S	Uncharacterized conserved protein, circularly permuted ATP-grasp superfamily	Uncharacterized putative protein	Function unknown
COG2188	K	DNA-binding transcriptional regulator, GntR family	Putative transcriptional repressor	DNA-binding transcriptional regulation
COG0583	K	DNA-binding transcriptional regulator, LysR family	HTH-type transcriptional regulator DmlR	Transcriptional regulator of dmlA (aerobic growth on d-malate as the sole carbon source)
COG1529	C	CO or xanthine dehydrogenase, Mo-binding subunit	Putative caffeine dehydrogenase subunit alpha	Oxidation-reduction process

a COG description of supposed target orthologous genes of regulatory region hits identified by 10 *k*-mers (*P* value 1.13 × 10^−4^) in the most competitive strains (Sinorhizobium meliloti GR4, KH35c, KH46, SM11).

10.1128/mSystems.00550-21.7 FIG S6 Distribution of 51 top *k*-mers (single plus mixed nodule occupancy) in Sinorhizobium meliloti AK58, GR4, KH35c, KH46, SM11, and RU11/001. (A to G) Distribution of orthologous gene hits among six S. meliloti strains (A) and distribution of orthologous genes among replicons of S. meliloti strains AK58 (B), GR4 (C), KH46 (D), KH35c (E), SM11 (F), and RU11/001 (G). (H to N) Distribution of unannotated CDSs among six S. meliloti strains (H) and distribution of unannotated CDSs among replicons of S. meliloti strains AK58 (I), GR4 (J), KH46 (K), KH35c (L), SM11 (M), and RU11/001 (N). (O to U) Distribution of putative regulatory region hits of orthologous gene hits among four S. meliloti strains (O) and among replicons of S. meliloti strains AK58 (P), GR4 (Q), KH46 (R), KH35c (S), SM11 (T), and RU11/001 (U). (V to A2) Distribution of regulatory region hits of unannotated CDSs among six S. meliloti strains (V) and among replicons of S. meliloti strains AK58 (W), GR4 (X), KH46 (Y), KH35c (Z), SM11 (A1), and RU11/001 (A2). Download FIG S6, TIF file, 1.7 MB. Copyright © 2021 Bellabarba et al. 2021 Bellabarba et al. https://creativecommons.org/licenses/by/4.0/ This content is distributed under the terms of the Creative Commons Attribution 4.0 International license.

Most of the genes associated with the 51 best *k*-mers belonged to the accessory genome, and only a few (nine genes) were within the core genome of the strains tested, suggesting a higher scoring of indel *k*-mers. All reported genes were also checked with SYMbiMICS (https://iant.toulouse.inra.fr/symbimics/) to examine their levels of transcription within nodules (33). As expected for most of the genes, we did not obtain any match because they were not present in the genome of S. meliloti 2011. However, the expression of all those genes that are shared with S. meliloti 2011 has been observed in different zones of the nodule (33), and depending on the gene, FI, FIId, and ZIII were the zones with higher levels of expression (see supplemental File S2A at https://doi.org/10.5061/dryad.x95x69pj5).

### Putative genetic determinants associated with increased competition and coinfecting nodule capabilities in assays versus S. meliloti BL225C.

Association analysis was also performed applying the competition matrix with the sum of single and mixed nodules (Table S2) as well as modeling analysis (see Text S1). Significantly associated-phenotype *k*-mers (*P* value < 0.05) were identified for each competition data set (see supplemental File S1B at https://doi.org/10.5061/dryad.x95x69pj5). Similar to the previous analysis, the set of *k*-mers related to competition versus BL225C showed the widest range of *P* values (Table 1). Among these, 51 top *k*-mers (*P* value = 5.14 × 10^−5^, Table S4b) were tracked down in the genomes of six strains that showed competition capabilities higher than 80% (evaluated as the nodule occupancy of single and mixed nodules; S. meliloti GR4, SM11, KH35c, KH46, AK58, and RU11/001; see supplemental File S1B at https://doi.org/10.5061/dryad.x95x69pj5, highlighted in bold). Fifty of the 51 top *k*-mers were mapped on 202 CDSs (see supplemental File S2B at https://doi.org/10.5061/dryad.x95x69pj5). Among these 202 CDSs, 99 were orthologous gene hits. The presence of gene hits with annotated function was uniformly distributed in all six strains (Fig. S6A); they were mainly located on the symbiosis-required megaplasmid pSymA (ranging from 52.6 to 84.4%; Fig. S6C, D, E, F, and G). Thirty-three orthologous gene hits were identified in both association analyses performed (Table 4 and see supplemental File S2B at https://doi.org/10.5061/dryad.x95x69pj5, highlighted in bold). In particular, these common genes were located on the specific region of the symbiotic megaplasmid previously reported (Fig. 2A) and exclusively found in the genomes of GR4, KH35c, and KH46 (Fig. 2A and see supplemental File S2B at https://doi.org/10.5061/dryad.x95x69pj5). Consequently, “common” candidate functions were outlined by both sets of *k*-mers as linked to the competition phenotype ascertained in GR4, KH35c, and KH46 (Table 4). Moreover, additional genes located in the same genomic region, which were not retrieved among the top *k*-mers in the single nodule occupancy data set, were found (Fig. 2A).

**TABLE 4:** List of functions putatively involved in promoting competition capabilities and nodule coinfection^*a*^

COG ID	COG class(es)	COG functional category	Prokka annotation/other annotation	Biological process
COG0154	J	Asp-tRNAAsn/Glu-tRNAGln amidotransferase A subunit or related amidase	Acylamidase	Aminoacyl-tRNA biosynthesis (glutaminyl-tRNAGln and l-asparaginyl-tRNAAsn)
COG0156	H	7-Keto-8-aminopelargonate synthetase or related enzyme	8-Amino-7-oxononanoate synthase	Biotin biosynthetic process
COG0242	J	Peptide deformylase	Peptide deformylase	Protein biosynthesis (cotranslational protein modification)
COG0376	P	Catalase (peroxidase I)	Catalase-peroxidase KatG	Hydrogen peroxide catabolic process; response to oxidative stress
COG0391*	GH	Archaeal 2-phospho-l-lactate transferase/bacterial gluconeogenesis factor, CofD/UPF0052 family	Putative phosphoenolpyruvate transferase FbiA	Coenzyme F_420_ biosynthesis
COG0410*	E	ABC-type branched-chain amino acid transport system, ATPase component	High-affinity branched-chain amino acid transport ATP-binding protein LivF	High-affinity branched-chain amino acid transport
COG0418*	F	Dihydroorotase	Dihydroorotase PyrC	Pyrimidine nucleotide biosynthesis
COG0494	V	8-Oxo-dGTP pyrophosphatase MutT and related housecleaning NTP	GDP-mannose pyrophosphatase NudK/nudix-type nucleoside diphosphatase (YffH/AdpP family)	Nucleoside phosphate metabolic process; ribose phosphate metabolic process
COG0507	L	ATP-dependent exoDNase (exonuclease V), alpha subunit, helicase superfamily I	ATP-dependent RecD-like DNA helicase	Recombinational DNA repair
COG0516	F	IMP dehydrogenase/GMP reductase	Inosine-5′-monophosphate dehydrogenase	Purine metabolism
COG0559	E	Branched-chain amino acid ABC-type transport system, permease component	High-affinity branched-chain amino acid transport system permease protein LivH	Branched-chain amino acid transport
COG0582	L	Integrase	Tyrosine recombinase XerC	Chromosome segregation; DNA recombination
COG0630	U	Type IV secretory pathway ATPase VirB11 (archaellum biosynthesis ATPase)	Type IV secretion system protein VirB11	Protein transport; infection
COG0683*	E	ABC-type branched-chain amino acid transport system, periplasmic component	Leucine-, isoleucine-, valine-, threonine-, and alanine-binding protein Brac	Branched-chain amino acid transport
COG1024*	I	Enoyl-CoA hydratase/carnitine racemase	Putative fatty acid oxidation complex subunit alpha–enoyl-CoA hydratase FadJ	Fatty acid metabolism
COG1028	IQR	NAD(P)-dependent dehydrogenase, short-chain alcohol dehydrogenase family	Putative dihydroanticapsin 7-dehydrogenase	Short-chain dehydrogenase
COG1060*	H	2-Iminoacetate synthase ThiH (tyrosine cleavage enzyme, thiamine biosynthesis)	FbiC FO synthase	Coenzyme F_0_ biosynthesis
COG1086	MO	NDP-sugar epimerase, includes UDP-GlcNAc-inverting 4,6-dehydratase FlaA1 and capsular polysaccharide biosynthesis protein EpsC	UDP-*N*-acetyl-alpha-d-glucosamine C6 dehydratase	Protein glycosylation
COG1137	M	ABC-type lipopolysaccharide export system, ATPase component	Lipopolysaccharide export system ATP-binding protein LptB	Lipopolysaccharide transmembrane transport
COG1171*	E	Threonine dehydratase	Diaminopropionate ammonia-lyase	Cellular amino acid catabolic process
COG1172	G	Ribose/xylose/arabinose/galactoside ABC-type transport system, permease component	Ribose import permease protein RbsC	Ribose transmembrane transport
COG1176	E	ABC-type spermidine/putrescine transport system, permease component I	Putrescine transport system permease protein PotH	Putrescine transport
COG1228	Q	Imidazolonepropionase or related amidohydrolase	Putative imidazolonepropionase	Nucleotide metabolism
COG1282*	C	NAD/NADP transhydrogenase beta subunit	NAD(P) transhydrogenase subunit beta PntB	Oxidation-reduction process; nicotinate and nicotinamide metabolism
COG1317	NU	Flagellar biosynthesis/type III secretory pathway protein FliH	Type III secretion protein L	Protein transport; infection
COG1478	H	F_420-0_:gamma-glutamyl ligase (F_420_ biosynthesis)	Bifunctional F_420_ biosynthesis protein FbiB	Coenzyme F_420_ biosynthesis (factor 420 polyglutamylation)
COG1529*	C	CO or xanthine dehydrogenase, Mo-binding subunit	Putative caffeine dehydrogenase subunit alpha	Oxidation-reduction process
COG1629	P	Outer membrane receptor proteins, mostly Fe transport	Heme/hemopexin utilization protein C	Siderophore transmembrane transport
COG1793	L	ATP-dependent DNA ligase	Multifunctional nonhomologous end joining protein LigD	DNA damage and repair
COG1920	H	2-Phospho-l-lactate guanylyltransferase, coenzyme F_420_ biosynthesis enzyme	Phosphoenolpyruvate guanylyltransferase FbiD	Coenzyme F_420_ biosynthesis
COG2081	R	Predicted flavoprotein YhiN	Putative protein/predicted flavoprotein (HI0933-like protein)	Unknown function
COG2898	S	Lysylphosphatidylglycerol synthetase, C-terminal domain, DUF2156 family	Putative phosphatidylglycerol lysyltransferase	Lipid metabolism
COG2948	U	Type IV secretory pathway, VirB10 components	Type IV secretion system protein VirB10	Protein transport; infection
COG3288*	C	NAD/NADP transhydrogenase alpha subunit	NAD(P) transhydrogenase subunit alpha PntA	Oxidation-reduction process; nicotinate and nicotinamide metabolism
COG3451	U	Type IV secretory pathway, VirB4 component	Type IV secretion system protein VirB4	Protein transport; infection
COG4178	R	ABC-type uncharacterized transport system, permease and ATPase components	Putative inner membrane ABC transporter ATP-binding protein YddA	Lipid transport

a COG description of gene hits identified by 50 top *k*-mers (*P* value 5.14 × 10^−5^) in the most competitive strains as single occupants and coinhabitants of mixed nodules (Sinorhizobium meliloti GR4, SM11, KH35c, KH46, AK58, and RU11/001). Function/annotation are reported according to the annotation performed with Prokka in this work and/or using original annotation. Asterisks indicate orthologous gene hits identified in both association analyses.

**FIG 3:**
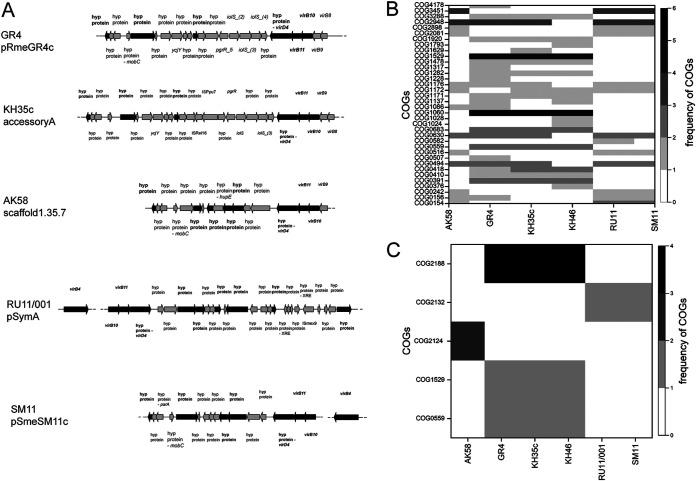
Genetic determinants associated with single and mixed nodule occupancy. (A) *k*-mers mapping in a region containing a *vir* operon on the genomes of Sinorhizobium meliloti GR4, KH35c, AK58, RU11/001, and SM11. Genes containing one or more *k*-mers are indicated with a black arrow; gene annotation is referred to the Prokka output. (B and C) Frequency of candidate functions of gene hits (B) and regulatory regions (C) identified by 51 top *k*-mers in the most competitive strains. The frequency of candidate functions reported as COG annotations (rows) in each strain (columns) is represented by grayscale shades.

Similar to previous results, the frequency of identified candidate functions was dissimilar among the six strains (Fig. 3A). The most represented orthologous gene groups were related to the type IV secretion system (T4SS) (specifically VirB10 and VirB11 proteins) and the nucleoside phosphate metabolic process, which are common to all strains except for KH46 (Fig. 3A and B; Table 4). The species S. meliloti is characterized by the presence of different T4SSs. In particular, in S. meliloti KH35c, three T4SSs are present: a, b, and f (31). The organization of *vir* genes in the operon, identified in this work, was characterized by the presence of a homolog of *virD4* (Fig. 3A) and the lack of *virG/virF* genes (typical of T4SSb) and *rctA* (which is typical of T4SSa, which is common in all S. meliloti strains). Therefore, the *vir* genes identified were probably related to the T4SSf family. Candidate functions related to putrescine transport were also observed in all strains except for KH46. The *k*-mers related to type IV secretion system protein VirB4 were tracked only in AK58, RU11/001, and SM11 strains (Fig, A; Table 4). Besides, these three strains (AK58, RU11/001, and SM11) showed similar distribution profiles of candidate functions (Fig. 3B), with the exclusive presence of orthologous genes related to aminoacyl-tRNA biosynthesis and protein biosynthesis, as well as purine and lipid metabolism (Fig. 3B; Table 4). Several other functions were exclusively detected in single strains only (see Text S1).

Predicted protein-coding sequences (CDSs) with no assigned function, picked by the 50 top *k*-mers, were also identified (see supplemental File S2B at https://doi.org/10.5061/dryad.x95x69pj5), mostly in the AK58, RU11/001, and SM11 genomes (Fig. S6H). The distribution of these CDSs among strain replicons was different compared to the single nodule occupancy results (Fig. S5 and S6). In strains SM11 and RU11/001, a large part of the CDS with no assigned function was located on symbiosis-required megaplasmid pSymA (Fig. S6M and N), whereas in GR4, KH46, and KH35c, most of the CDSs were also located on the chromosome and other accessory replicons (Fig. S5J, K, and L).

Fourteen of the 51 top *k*-mers analyzed pinpointed 29 regulatory regions hit (Fig. 3C and Table 5; see also supplemental File S3B at https://doi.org/10.5061/dryad.x95x69pj5). Among these, 12 regulatory region hits on putatively orthologous gene targets were identified, which were located on pSymA in all S. meliloti strains (Fig. S6Q to U). Six of them were found in both association analyses performed in the genomes of GR4, KH35c, and KH46 (see supplemental File S3B at https://doi.org/10.5061/dryad.x95x69pj5). Seventeen regulatory regions were associated with CDS targets with no assigned function (see supplemental File S3B at https://doi.org/10.5061/dryad.x95x69pj5) and tracked to a greater extent in RU11/001, SM11, KH46, and AK58 (Fig. S6V). The distribution of these regulatory regions among replicons was not similar among strains (Fig. S6). In strains GR4 and KH35c, all regulatory region hits were located on the chromosome (Fig. S6X and Z), whereas in KH46, they were mainly found on pSymB (Fig. S6Y), and in RU11/001 and SM11, they were found on pSymA (Fig. S6A1 and B1).

**TABLE 5:** List of regulatory regions putatively involved in promoting competing abilities and capabilities of coinfecting nodules^*a*^

COG ID	COG class(es)	COG functional category	Prokka annotation/product	Biological process(es)
COG2132	DMP	Multicopper oxidase with three cupredoxin domains (includes cell division protein FtsP and spore coat protein CotA)	Putative blue copper oxidase CueO	Detoxification of copper ion
COG2188	K	DNA-binding transcriptional regulator, GntR family	Putative transcriptional repressor	DNA-binding transcriptional regulation
COG0559	E	Branched-chain amino acid ABC-type transport system, permease component	High-affinity branched-chain amino acid transport system permease protein LivH	Branched-chain amino acid transport
COG1529	C	CO or xanthine dehydrogenase, Mo-binding subunit	Putative caffeine dehydrogenase subunit alpha	Oxidation-reduction process
COG2124	QV	Cytochrome P450	Putative pentalenolactone synthase (cytochrome P450 CYP2 subfamily)	Pentalenolactone biosynthetic process

a COG description of supposed target orthologous genes of regulatory region hits identified by 14 top *k*-mers (*P* value 5.14 × 10^−5^) in the most competitive strains as single occupants and coinhabitants of mixed nodules (Sinorhizobium meliloti GR4, SM11, KH35c, KH46, AK58, and RU11/001).

## DISCUSSION

Rhizobium-legume symbiosis is a paradigmatic example of a bacterium-plant association. The ability to colonize plant tissue is under selective pressure, and rhizobial strains which efficiently colonize host plants can more effectively promote plant growth, giving rise to a partial “fitness alignment” between the host and the symbiont (34, 35). However, in nature, several strains compete for forming symbiotic associations with the host plant, and often, nodules are simultaneously colonized by different strains, which in turn may have different efficiencies in promoting plant growth; some of them also behave as “cheaters” (7, 14, 18, 36). In this sense, measuring the competitiveness for plant and nodule colonization and predicting this phenotype from rhizobial genome sequences are of paramount importance for understanding the evolution of rhizobium-plant symbiosis and developing effective inoculant strains for increasing agricultural yields of legume crops (7).

In a recent work, the first step of symbiotic colonization was elucidated by analyzing gene expression patterns related to the response of root exudates among different rhizobial strains and plant varieties (37). Here, we addressed the possibility of identifying some of the genetic determinants involved in strain competitiveness for symbiotic nodule formation (the functional-symbiotic structure). The host plant-rhizobial symbiont system of alfalfa and S. meliloti was employed, and direct measurements of competitiveness were obtained through the analysis of nodule occupancy. This experimental design pointed out a wide variety of strain responses to the diverse competitive conditions, identifying three different competition patterns and outlining a highly complex phenotype that strongly depends on the engaged competitor. The strains used in this work were originally isolated from different *Medicago* species. However, they displayed good competition capabilities in nodulation of alfalfa, indicating that nodulation competitiveness is not strictly bound to the host genotype.

An abundant presence of mixed nodules was also observed in all three sets of experiments, confirming previous results (11, 13, 37) and suggesting that the possibility to coinfect nodules by different strains could be more widespread than expected.

Strains M270 and T073 were characterized by low nitrogen fixation rates and showed low competition capabilities. Conversely, the differential responses of strains with medium-high nitrogen-fixation efficiency advances the idea that a greater competitive ability is not correlated with a high nitrogen fixation efficiency in alfalfa–S. meliloti interaction, as previously suggested (38, 39). Concerning the competition versus BL225C, except for GR4 and SM11, this assumption seemed to be particularly true for highly efficient N-fixer strains (USDA1157, CCMM B554, RU11/001, 2011, and AK58), which turned out to be medium-weak competitors.

Subsequently, a method based on *k*-mers was used for the evaluation of the genetic determinants responsible for an increased competition phenotype. The number of GWASs has progressively increased in recent years (20, 40, 41) because of the flexibility in capturing different types of genetic variants and overcoming the alignment of sequences to a reference genome. Among the different pipelines adopted for this kind of analysis, PhenotypeSeeker is one of the up-to-date tools that use machine learning for predicting phenotypes from the sole genome sequences (32). The *k*-mers related to the competition versus BL225C (single nodule occupancy) taken into account were significantly correlated with the phenotype of interest and mapped on the genomes of the four most competitive strains (GR4, KH35c, KH46, and SM11). Therefore, they may be considered the most informative *k*-mers for tagging the genetic variants associated with remarkable competition capabilities. The same approach was used considering the sum of single and mixed nodules. In this case, the group formed by highly competitive strains was larger and included strains AK58 and RU11/001. Moreover, 40% of the *k*-mers previously identified considering only single nodules could also be retrieved in this second analysis.

The largest part of the genes putatively associated with competitiveness was harbored by the megaplasmid pSymA (or pSymA homologs depending on the strain), which is the genomic element carrying all the genes necessary for symbiosis (e.g., *nod*, *fix*, and *nif* genes) (42). According to a previous study, pSymA harbors the largest part of genomic diversity in S. meliloti (43), largely contributing to the phenotypic diversity among strains; considering the obtained results, it may also be linked to competition capabilities.

Previous studies have highlighted the importance of exopolysaccharide production, motility, and signaling for responses to root exudates, symbiosis establishment, and competition (25, 27, 37). In the work of Burghardt et al., a variation in allele frequency for genes whose function is related to cell motility, nitrogen fixation, and nodule formation was observed (25). Conversely, the genes (with functional annotation) found in this work, putatively associated with competitiveness (for single nodule occupancy), were mostly related to biosynthesis and transport functions. Concerning the differences between our results and those from the study by Burghardt et al. (25), we should point out that the experimental settings were different. In the work of Burghardt et al. (25), a mix of 101 strains was used to inoculate plants, whereas we performed competition experiments with two strains at a time. Additionally, none of the strains used were common in the two studies. Concerning the association analysis, Burghardt et al. focused on single nucleotide polymorphisms (SNPs), identifying as responsible for variation in relative fitness those alleles showing the highest variation in frequency in response to selection (25). In our approach, we observed a stronger signal related to indels, with most of the genes retrieved belonging to the accessory genome. However, one SNP associated with the gene *htpG* (one of the genes with the strongest signal identified in the work of Burghardt et al. [25]) was also retrieved in our analysis by four *k*-mers, albeit with a lower *P* value (3.09 × 10^−04^). These four *k*-mers separate strains into two groups: one formed by 10 strains and the other one composed of strains T073, 2011, and USDA1157, which are the strains with the lowest competition capabilities in the assay versus BL225C. In both experiments, a sterilized substrate was used, and therefore, we need to consider that under soil conditions, the competition and the advantage in nodule entry may differ depending on the soil physical/chemical features and the presence of an indigenous microbiota.

Many *k*-mers were related to COG1060, which, together with COG0391, was linked to the presence of the *fbi* operon in strains KH46, KH35c, and GR4. The *fbi* operon is widely distributed in aerobic soil bacteria and is responsible for the synthesis of the functional versatile redox factor F_420_ (44, 45). This cofactor is involved in the redox modification of many organic compounds, facilitating low-potential two-electron redox reactions (44, 45). Moreover, the presence of this cofactor is linked to several important processes such as persistence, antibiotic biosynthesis (tetracyclines, lincosamides, and thiopeptides), and prodrug activation (46), possibly increasing the fitness of these strains. However, the role of this function over symbiotic competition deserves further attention.

Another group of COGs highly represented was related to ABC transporters. The S. meliloti genome encodes a large number of ABC uptake and export systems (47, 48). This feature is probably linked to the selective adaptation to oligotrophic soils (48, 49). According to our association analysis, a group of *k*-mers tagged putative genes encoding ATPase and permease subunits of branched-chain amino acid ABC transport complex Bra/Liv (50). In S. meliloti, a double mutant for the two main amino acid ABC transport complexes (*aap bra*) showed no reduction in N_2_ fixation efficiency and no influence on the plant phenotype, suggesting that in bacteroids, branched-chain amino acid auxotrophy, called “symbiotic auxotrophy,” does not occur (51). However, an attenuated competitive phenotype was found in S. meliloti mutated in the *livM* gene, which encodes the permease subunit of the Bra/Liv complex (52). It is therefore reasonable to suppose that this complex may provide a noteworthy benefit in the competition dynamics, ensuring a higher supply of amino acids under free-living rhizosphere conditions and increasing strain competitiveness (52, 53). Other COGs related to the ABC transporter were COG1129 and COG1172. Proteins grouping in COG1129 are ATPase components of an ABC-type ribose import system. In Rhizobium leguminosarum, a putative ribose ABC transporter (RbsA, RL2720) was induced by the presence of arabinogalactan, and it was specifically overexpressed in the alfalfa rhizosphere (50). The COG1172, detected by both association analyses, contains a ribose/xylose/arabinose/galactoside ABC-type transport system permease component, highlighting the importance of efficient carbon uptake in the rhizosphere to outcompete other bacteria. The COG1172 is also related to the import of autoinducer signaling molecules in the quorum sensing process, whose connection with S. meliloti competitive behavior has been reported (54).

The type IV secretion system was also found in our association analysis (T4SSf). Interestingly, T4SSa (the type commonly found in S. meliloti) is not required for symbiosis (55), whereas T4SSb has been linked to competitive advantage in S. meliloti for nodule occupancy in *M. truncatula* (56) and is associated with competition for rhizosphere colonization (57). We cannot exclude a role of T4SS in the management of host defenses, as suggested for Mesorhizobium loti (31, 58, 59). However, the role of T4SSf in the competition should be further elucidated. Several COGs were related to the metabolism of different compounds: pyrimidine (COG0418; dihydroorotase), glutamate (COG1788 and COG2057; glutaconate coenzyme A [CoA] transferase), amino acids (COG1171; threonine dehydratase), fatty acids (COG1024; enoyl-CoA hydratase/carnitine racemase), and glycerol (COG1028; glycerol dehydrogenase), reinforcing the importance of metabolic versatility for nodule colonization (60, 61). Testing those functions through metabolic model reconstructions of the different strains may clarify the importance of metabolism in the competition for nodule colonization (62).

One group of *k*-mers fell within transcriptional regulation genes, suggesting their involvement in a fine-tuning bacterial response to the presence of other competitors and/or a quick response to variations in the external conditions.

Other COGs retrieved had less-clear connections with competitiveness, and it will require further studies to infer their possible role in this process.

A substantial part of *k*-mers mapped on hypothetical genes with unknown functions. Although many genes required for rhizobial adaptation to the rhizosphere are not yet characterized, transcriptomic analysis of rhizobia isolated from the rhizosphere revealed the expression of many hypothetical genes (49). This finding suggests that in the pangenome of S. meliloti, several functions potentially important in the fitness associated with the symbiotic interaction, and possibly in plant growth promotion, are yet to be discovered. However, the list of candidate genes (hypothetical and not) identified in this work needs to be experimentally validated to confirm their effectiveness in competition and to understand how their function influences strain competitiveness.

Most of the identified *k*-mers mapped on genes belonging to the accessory genome, and only a few genes of these lists were common with S. meliloti 2011 genes. This strain has been used for transcriptomic analysis within nodules, applying laser capture microdissection coupled to transcriptome sequencing (RNAseq) (33). Thanks to the SYMbiMICS website, we observed that these common genes were expressed within all different zones of the nodule infected by S. meliloti 2011 (33), indicating that they may play an active role in the symbiotic process.

Rhizobial competitiveness is a cornerstone for plant colonization, making the selection of highly competitive rhizobia fundamental for sustainable agricultural production. Here, we report the feasibility and reliability of using a *k*-mer-based GWAS approach to detect genes associated with this complex quantitative phenotype in the plant symbiont S. meliloti. Several functions possibly contribute to ameliorate competitiveness, indicating that many different bricks, increasing rhizobial versatility, pave the way for success in competition. This approach may provide the basis for a large-scale screening of putative competitiveness capabilities among pairs of strains, based on genome sequences. Interestingly, the evidence that most of the genes putatively associated with competition reside on the megaplasmid pSymA can offer the possibility to extend the creation of *ad hoc* hybrid strains by mobilizing the pSymA megaplasmid from different hosts (63) to develop novel ameliorated inoculants (7).

## MATERIALS AND METHODS

### Bacterial strains, plasmids, and growth conditions.

The strains and plasmids used in this work are listed in Table S3 in the supplemental material. Escherichia coli strains were grown in liquid or solid Luria-Bertani (LB) medium (Sigma-Aldrich) at 37°C (64), supplemented with tetracycline (10 μg/ml). Sinorhizobium meliloti strains were cultured in broth or agar tryptone yeast (TY) medium with 0.2 g/liter CaCO_3_ at 30°C (65), supplemented with streptomycin (500 μg/ml in broth and agar media), rifampin (50 μg/ml), and tetracycline (1 μg/ml in liquid broth medium, 2 μg/ml in agar medium), when necessary.

10.1128/mSystems.00550-21.10 TABLE S3 Strains and plasmids used in this work. Download Table S3, DOCX file, 0.02 MB. Copyright © 2021 Bellabarba et al. 2021 Bellabarba et al. https://creativecommons.org/licenses/by/4.0/ This content is distributed under the terms of the Creative Commons Attribution 4.0 International license.

### Construction of S. meliloti fluorescently tagged strains.

The S. meliloti strains were tagged with green fluorescent protein (GFP) or red fluorescent protein (RFP). Donor E. coli S17-1 strains containing plasmid pHC60 (harboring a constitutively expressed GFP [66]) or pBHR mRFP (harboring a constitutively expressed RFP [67]) were used for biparental conjugations with rifampin-resistant derivative S. meliloti strains. Spontaneous rifampin-derivative S. meliloti strains were isolated by plating aliquots of 100 μl of cell suspension of 10^9^ cells on agar TY medium with rifampin (50 μg/ml). Conjugal transfer was performed as previously described (68), and GFP and RFP expression was verified for each single strain by fluorescence microscopy with a Leica DM L (Leica, Germany) equipped with an N plan oil-immersion objective (100×/1.25 oil).

### Competition assay.

*Medicago sativa* (cv. Maraviglia) plantlets were germinated and grown as described in the nodulation assay section (Text S1). The S. meliloti strains were grown at 30°C to the late exponential phase (optical density at 600 nm [OD_600_] = 0.6 to 0.8) in TY with opportune antibiotics. Subsequently, each culture was washed twice in nitrogen-free solution (69) and diluted to a final concentration of approximately 5 × 10^4^ CFU/ml. The inoculum mixtures were prepared with equal volumes of cellular suspensions of two different fluorescently tagged strains. A total of 39 competition experiments were set up (13 GFP-tagged strains × 3 RFP-tagged strains), and we assumed an equal amount of fluorescence emission by all strains used. Six plants for each competition experiment were inoculated with 1 ml of inoculum mixtures per seedling. After 4 weeks, nodule fluorescence was detected using a fluorescence stereomicroscope, Stereo Discovery V12 (Zeiss, Oberkochen, Germany), equipped with a charge-coupled-device (CCD) camera controlled by the AxioVision software for image acquisition. For each plant, we determined the total number of nodules and the numbers of green (occupied by GFP-tagged strain only), red (occupied by RFP-tagged strain only), and yellow (occupied by both strains) nodules. For nodule color determination, each nodule of each single plant was imaged with filters for GFP (Zeiss filter set 38HE; excitation 470/40, emission 525/50) and DsRed (Zeiss filter set 43HE; excitation 550/25 and emission 605/70). The obtained images were processed with the ImageJ software (70). Nodule occupancy was expressed as the ratio of the number of nodules (green, red, or mixed) to the total number of nodules present on the roots of each plant. For each competition, we calculated the mean of the nodule occupancy for the six replicates.

### Statistical analysis.

Statistical data analysis was performed with the RStudio software (71). The Shapiro test was applied to evaluate data distribution; analysis of variance (ANOVA) and Tukey’s *post hoc* tests or nonparametric Kruskal-Wallis and *post hoc* Dunn tests were performed using the *FSA* and *rcompanion* packages. The principal-component analysis (PCA) and PERMANOVA were performed using the competition mean values with the PAST software (72).

### PhenotypeSeeker analysis.

The single nodule occupancy and the mean value of the sum of the single occupied nodules and the mixed nodules of the strains, assessed in the three competition experiments, were converted into continuous matrices of equivalent values between 0 and 1 for each data set. The FASTA genome sequences of 13 strains and the obtained matrices were used as input to count all *k*-mers for each set of competition; *k*-mer length was set to 13 nucleotides. In the first filtering step, the *k*-mers that were present in or missing from fewer than two samples (“–min 2–max 2”; default) were rejected. Clonal population structure correction was performed. The *k*-mers were tested for the analyses of association with the phenotype according to the weighted Welch two-sample *t* test, and *k*-mers with a *P* value higher than 0.05 were automatically discarded. Linear regression models were achieved using 1,000 top *k*-mers, with the lowest *P* value, for all three data sets. We applied 3-fold nested cross-validation, in which three different random combinations of training and test sets were used, and the model evaluation metrics were averaged over 3-fold train/test splits. The 3-fold explicitly indicates that each strain was included once into the test set and twice into the training set.

Nodulation and acetylene reduction assays and annotation and phylogenetic analyses, as well as mapping procedures, are reported in Text S1.
