# Supplementary figures and images for: Competitiveness for Nodule Colonization in Sinorhizobium meliloti: Combined In Vitro-Tagged Strain Competition and Genome-Wide Association Analysis
Source: mSystems. 2021 Jul 27;6(4):e00550-21. doi: 10.1128/mSystems.00550-21 (PMC8407117; doi:10.1128/mSystems.00550-21)

A

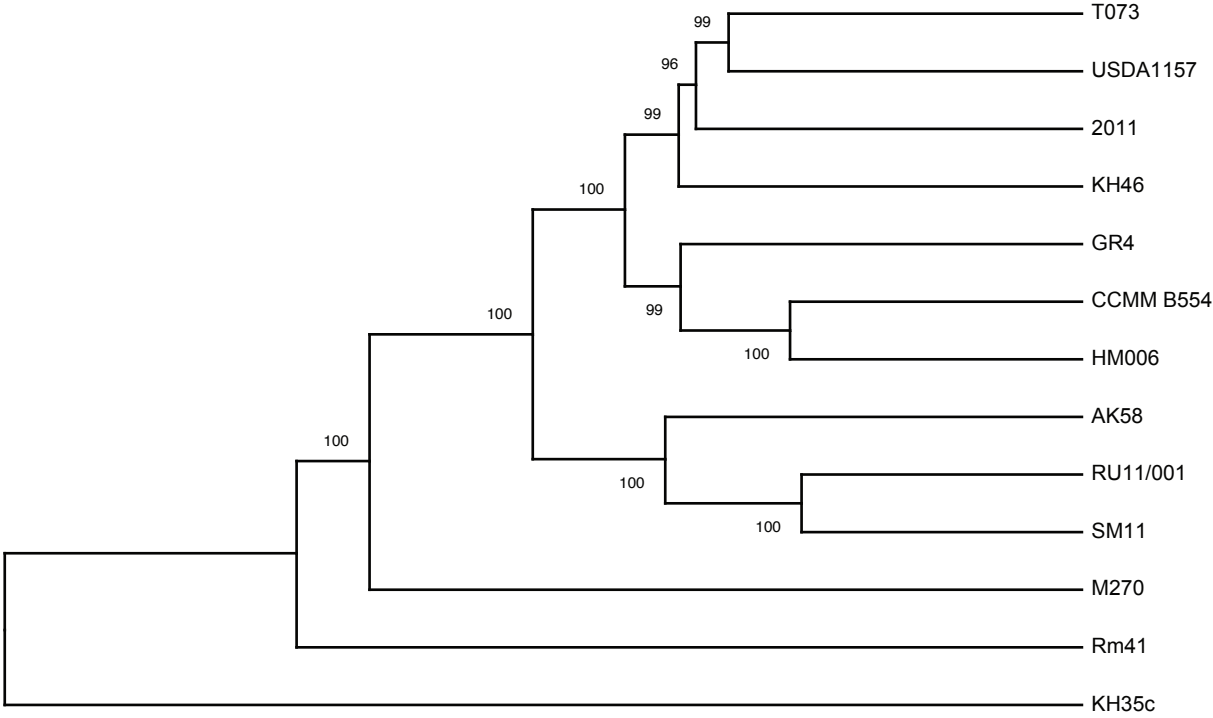

B

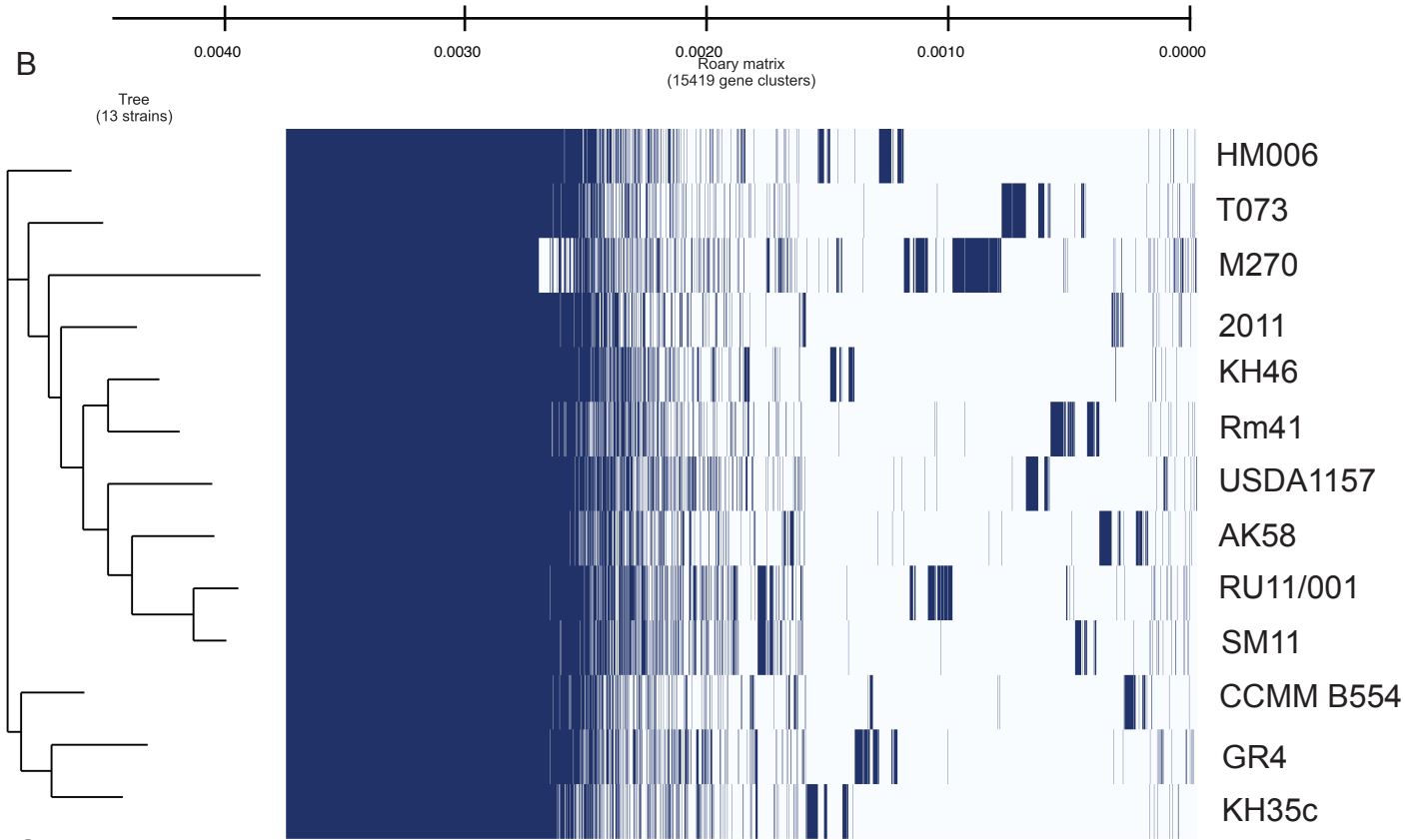

C

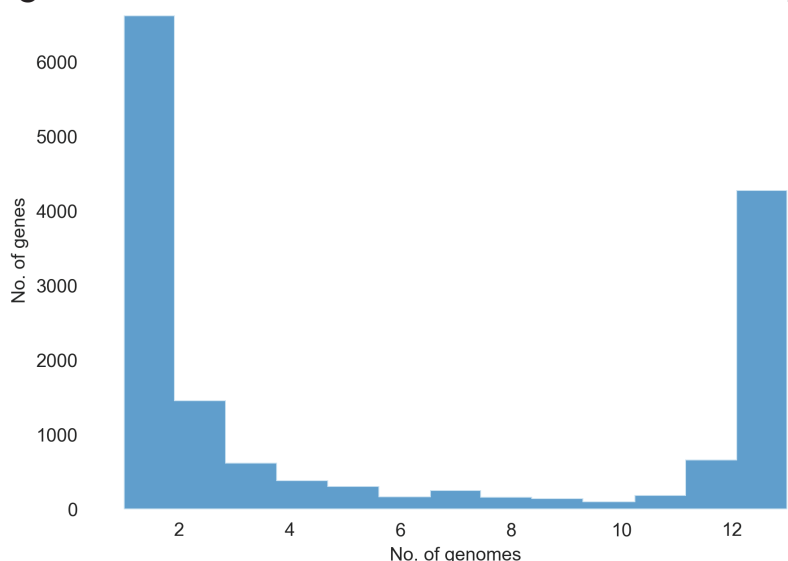

D

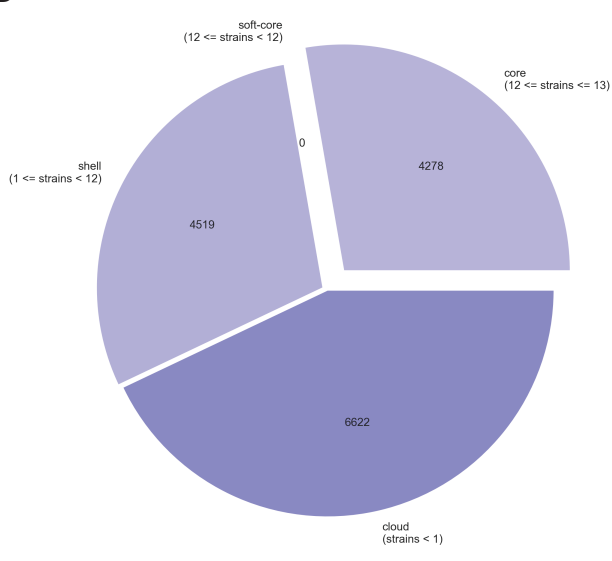

Supplement: FIG S1 [file msystems.00550-21-sf001.pdf]

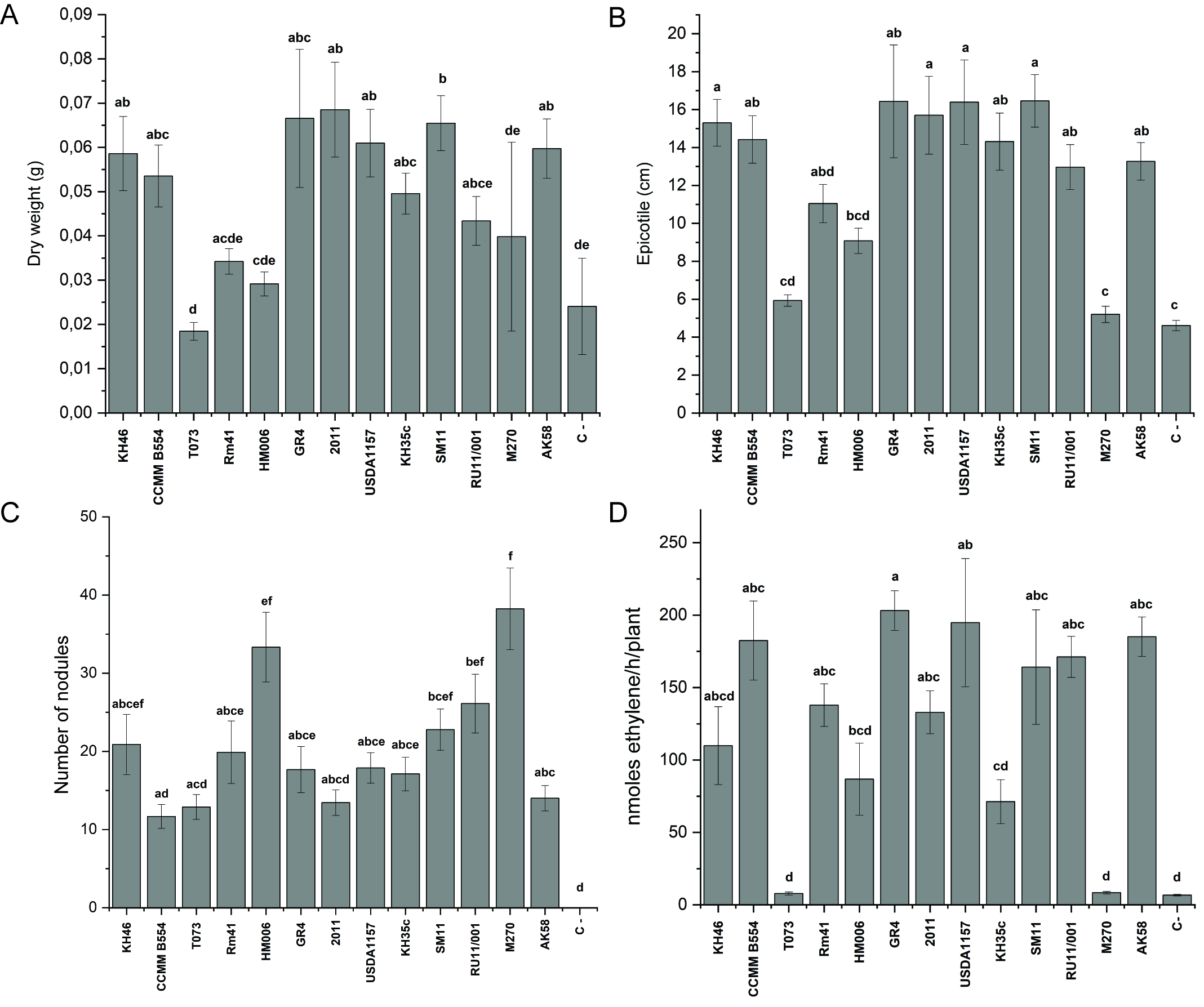

Supplement: FIG S2 [file msystems.00550-21-sf002.tif]

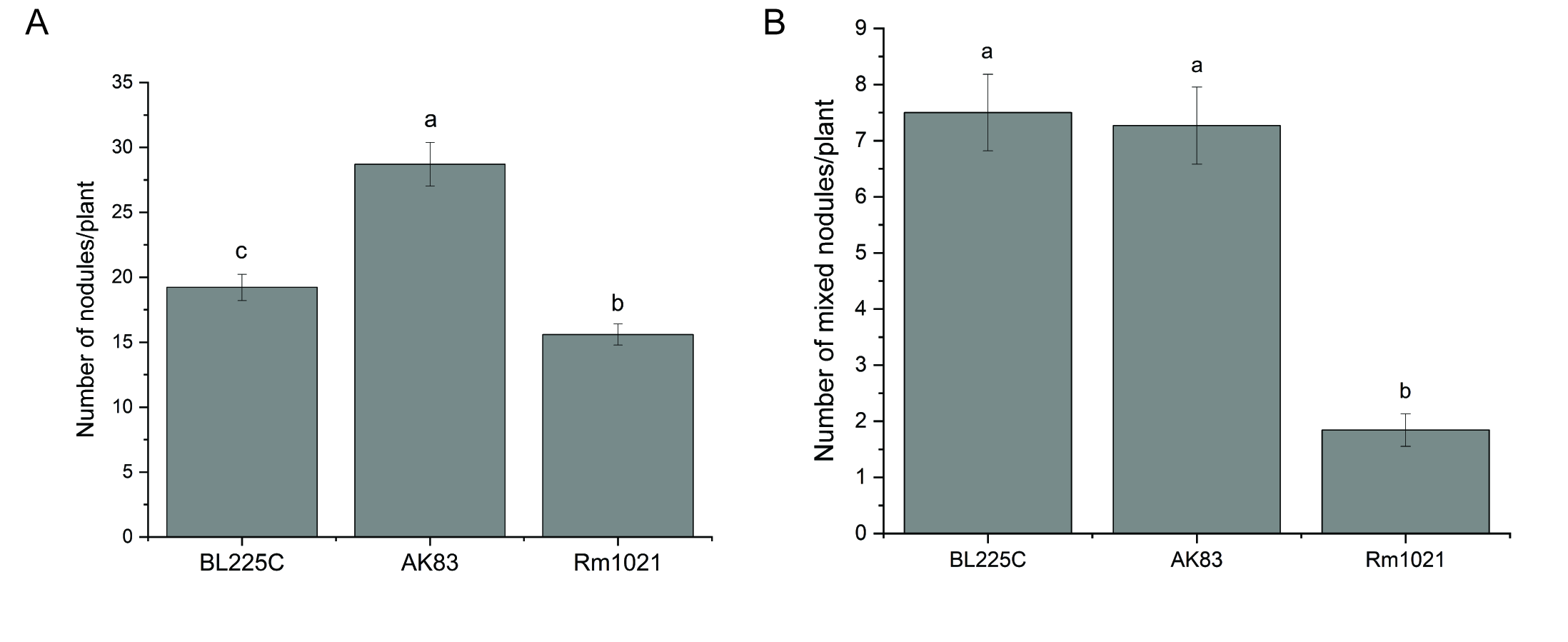

Supplement: FIG S3 [file msystems.00550-21-sf003.tif]

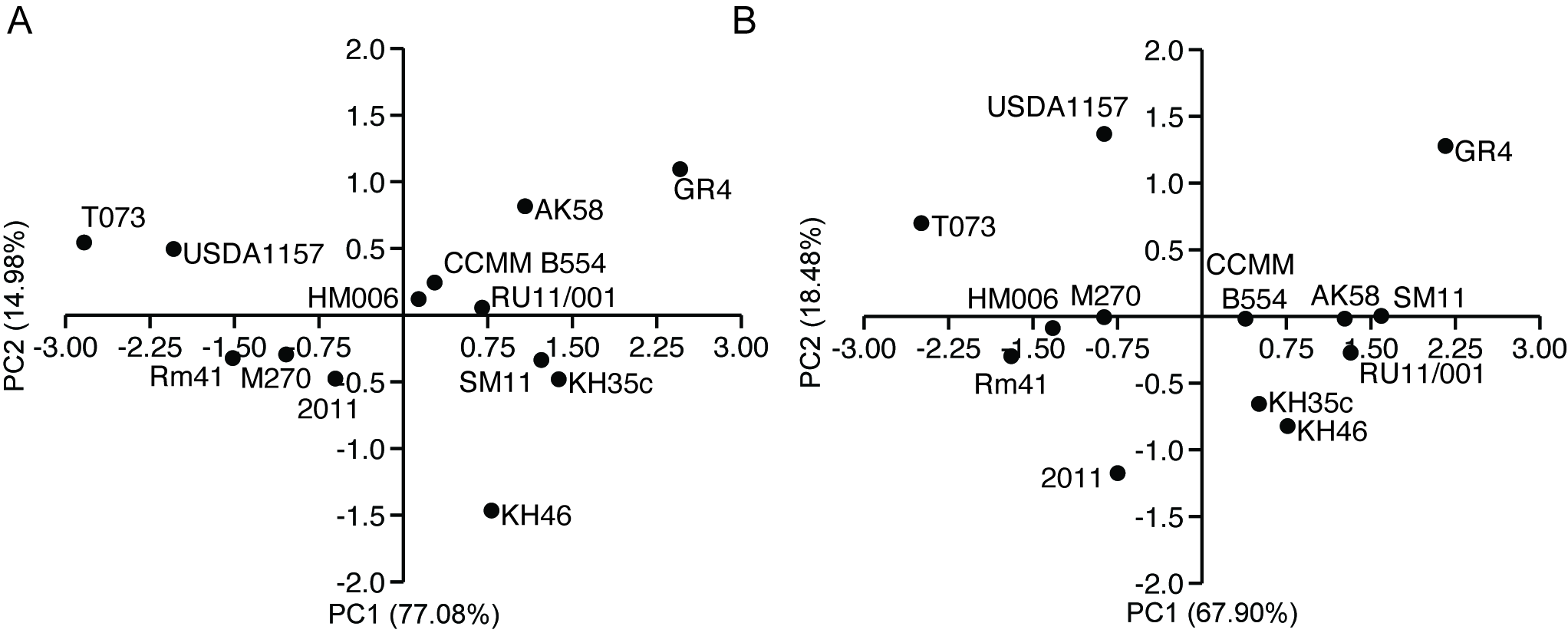

Supplement: FIG S4 [file msystems.00550-21-sf004.tif]

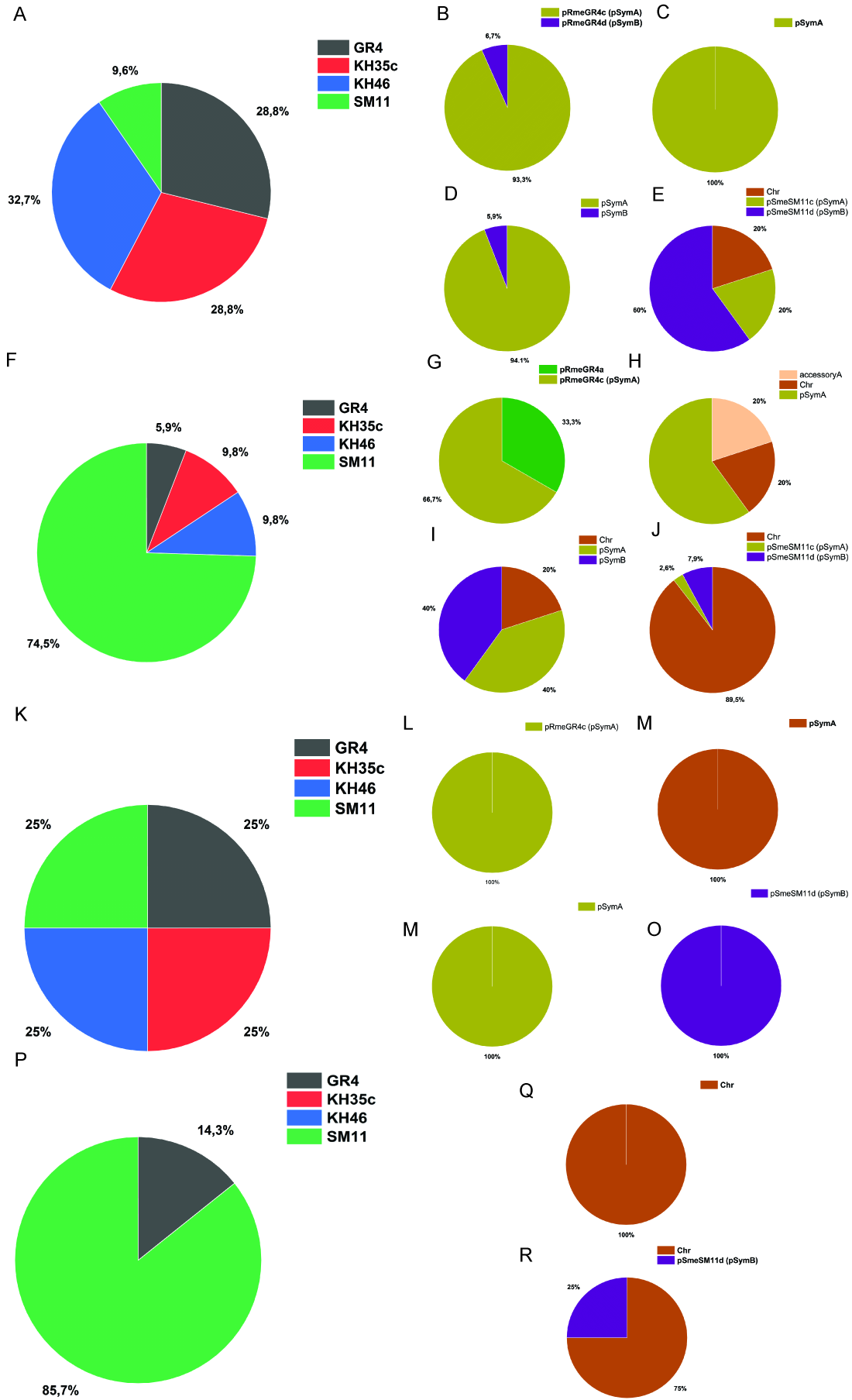

Supplement: FIG S5 [file msystems.00550-21-sf005.tif]

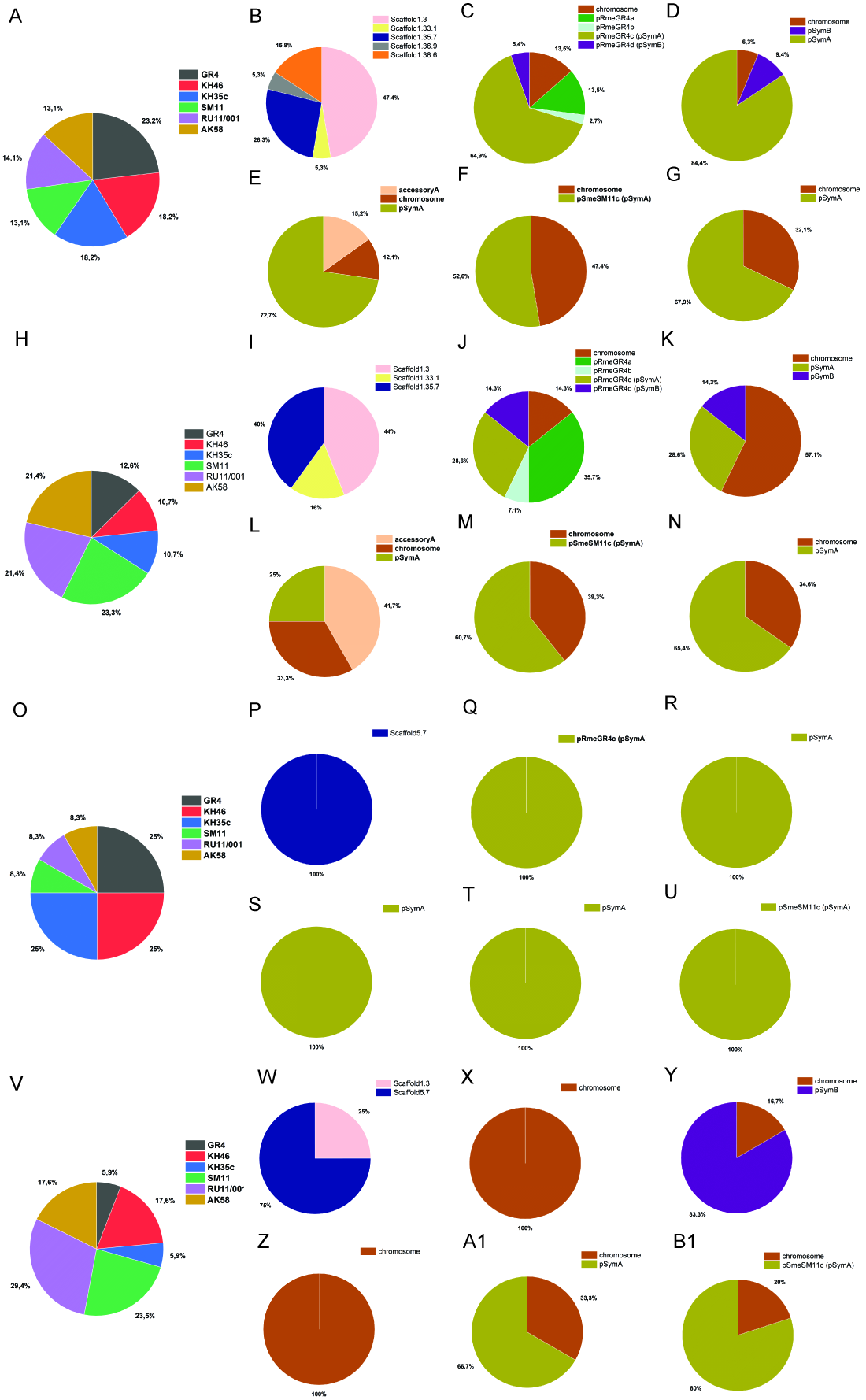

Supplement: FIG S6 [file msystems.00550-21-sf006.tif]
