# Supplementary material for: Competitiveness for Nodule Colonization in Sinorhizobium meliloti: Combined In Vitro-Tagged Strain Competition and Genome-Wide Association Analysis
Source: mSystems. 2021 Jul 27;6(4):e00550-21. doi: 10.1128/mSystems.00550-21 (PMC8407117; doi:10.1128/mSystems.00550-21)
Supplement: TABLE S1 [file msystems.00550-21-st001.docx]

**Table S1**. *Sinorhizobium meliloti* strains used in this work.

| **Strains** | **Source/Description** | **Genbank assembly codes** | **Reference** |
| --- | --- | --- | --- |
| AK83 | Geographic location: Kazakhstan; Host: *Medicago falcata* | GCA_000147795.3 (7) | (8) |
| 1021 | SU47 *str*-21 | GCA_000006965.1 (9) | (10) |
| BL225C | Geographic location: Italy; Host: *Medicago sativa* | GCA_000147775.3 (7) | (11) |
| KH46 | Geographic location: France; Host: *Medicago truncatula* | GCF_002197465.1 (12, 13) | (12) |
| CCMM B554 | Geographic location: Morocco; Host: *Medicago arborea* | GCA_002215195.1 (14) | (15) |
| T073 | Geographic location: Tunisia; Host: *Medicago truncatula* | GCA_002197145.1 (12, 13) | (12) |
| Rm41 | Geographic location: Hungary; Host: *Melilotus/Medicago* | GCA_000304415.1 (16) | (17) |
| HM006 | Geographic location: France; Host: *Medicago truncatula* | GCA_002197165.1 (12, 13) | (12) |
| GR4 | Geographic location: Spain ; Host: agricultural field | GCA_000320385.2 (18) | (18) |
| 2011 | SU47 | GCA_000346065.1 (19) | - |
| USDA1157 | Geographic location: USA, California; Host: *Medicago sativa* | GCF_002197025.1 (13) | (13) |
| KH35c | Geographic location: France; Host: *Medicago truncatula* | GCA_002197105.1 (12, 13) | (12) |
| SM11 | Geographic location: Germany; Host: agricultural field | GCA_000218265.1 (20) | (21) |
| RU11/001 | Geographic location: Germany; Host: *Medicago sativa* | GCA_001050915.2 (22) | (23) |
| M270 | Geographic location: Jordan; Host: *Medicago truncatula* | GCA_002197085.1 (12) | (12) |
| AK58 | Geographic location: Kazakhstan; Host: *Medicago falcata* | GCA_000473425.1 (24) | (8) |
