# Supplementary material for: Competitiveness for Nodule Colonization in Sinorhizobium meliloti: Combined In Vitro-Tagged Strain Competition and Genome-Wide Association Analysis
Source: mSystems. 2021 Jul 27;6(4):e00550-21. doi: 10.1128/mSystems.00550-21 (PMC8407117; doi:10.1128/mSystems.00550-21)
Supplement: TABLE S2 [file msystems.00550-21-st002.docx]

**File S2.** **Nodule occupancy.** Single nodule occupancy and sum of single and mixed nodules (in brackets) of *Sinorhizobium meliloti* tested strains in the competition experiments *vs* *S. meliloti* strains Rm1021, AK83 and BL225C. Different letters indicate differences (Kruskal-Wallis and Dunn test, *P* value < 0.05) within a competition assay (columns; *vs* BL225C, *vs* AK83, *vs* Rm1021).

| **Strains** | **vs BL225C** | | **vs AK83** | **vs Rm1021** |
| --- | --- | --- | --- | --- |
| AK58  CCMM B554  GR4  HM006  KH35c  KH46  M270  2011  Rm41  RU11/001  SM11  T073  USDA 1157 | 41.2% ^abcd^ (90.2% ^abc^)  42.9% ^abc^  (72.7% ^abd^)  66.7% ^a^ (89.3% ^abc^)  46.4% ^ab^ (54.0% ^d^)  68.3% ^a^ (88.9% ^abc^)  68.9% ^a^ (93.4% ^ac^)  37.5% ^abcd^ (74.5% ^abd^)  15.5% ^bcd^ (67.3% ^bd^)  13.9% ^bcd^ (62.3% ^bd^)  39.4% ^abcd^ (97.9% ^c^)  63.4% ^a^ (97.6% ^c^)  0.4% ^d^ (65.1% ^bd^)  8.3% ^cd^ (78.1% ^abd^) | 45.7% ^a^ (58.7% ^ab^)  28.6% ^ab^ (56.5% ^ab^)  63.9% ^a^ (86.0% ^a^)  25.2% ^ab^ (42.7% ^b^)  28.4% ^abc^ (41.8% ^b^)  7.0% ^bc^ (40.9% ^b^)  6.8% ^bc^ (38.2% ^b^)  8.6% ^bc^ (31.0% ^b^)  1.7% ^c^ (30.5% ^b^)  30.0% ^ab^ (52.2% ^ab^)  29.1% ^ab^ (59.2% ^ab^)  1.8% ^c^ (28.9% ^b^)  9.1% ^bc^ (51.4% ^ab^) | | 82.7 % ^cd^ (100.0 % ^c^)  65.0 % ^acd^ (95.8 % ^cd^)  93.4 % ^d^ (97.3 % ^cd^)  58.6 % ^abcd^ (71.6 % ^abd^)  89.3 % ^d^ (94.5 % ^bcd^)  90.0 % ^d^ (100.0 % ^c^)  37.0 % ^abc^ (58.8 % ^ab^)  84.8 % ^d^ (96.1 % ^cd^)  50.5 % ^abcd^ (56.5 % ^abd^)  89.0 % ^d^ (98.2 % ^c^)  86.6 % ^d^ (100.0 % ^c^)  0.00 % ^b^ (9.3 % ^a^)  19.7 % ^ab^ (24.5 % ^a^) |
