# Supplementary material for: Competitiveness for Nodule Colonization in Sinorhizobium meliloti: Combined In Vitro-Tagged Strain Competition and Genome-Wide Association Analysis
Source: mSystems. 2021 Jul 27;6(4):e00550-21. doi: 10.1128/mSystems.00550-21 (PMC8407117; doi:10.1128/mSystems.00550-21)
Supplement: TEXT S1 [file msystems.00550-21-t0001.docx]

**Supplementary results and discussion**

**Putative genetic determinants associated with increased competition and co-infecting nodule capabilities in assays *vs* *S. meliloti* BL225C. Functions detected in single strains only.** Functions related to recombinational DNA repair (COG0507), nucleotide metabolism (COG1228), and lipopolysaccharide and lipid transmembrane transport (COG1137, COG4178) were founded in GR4 (Fig 3B, Table 4). Interestingly, a putative type III secretion protein L (COG1317) was pinpointed solely in the GR4 genome (Fig. 3B, Table 4). Likewise, some orthologous genes hits were tagged in the KH46 genome, as a putative DNA ligase involved in DNA damage and repair process (COG1793) (Fig. 3B, Table 4). A putative catalase-peroxidase KatG (COG0376), involved in response to oxidative stress, and a putative NAD(P)-dependent short-chain alcohol dehydrogenase (COG1028), with unknown function, were exclusively found in KH46 (Fig. 3B, Table 4). Exclusive orthologous genes as tyrosine recombinase XerC (COG0582), involved in DNA recombination, and a putative protein C (COG1629), involved in siderophore transmembrane transport, were detected in RU11 and KH35c strains respectively (Fig. 3B, Table 4).

**Modeling competition pattern from genome sequences.** Statistical models for competitive phenotype prediction were built within the PhenotypeSeeker workflow considering both single and single plus mixed nodules occupancy. Afterward *k*-mers selection, linear regression models were trained for each set of competition experiments in both the association analysis. The phenotype predictabilities were evaluated by splitting data into three different random combinations of training and test sets, then averaging the model evaluation metrics over these splits. Using this 3-fold nested cross-validation, each strain was once included in the test set and twice included in the training set. This approach aims to improve the precision of generalization performance estimation, which may suffer from limited test set size if only a single split is used.

The effectiveness of linear regression models in describing the three observed competition patterns varied among the datasets. For single nodule occupancy, the prediction was accurate in the competition versus BL225C and AK83, as indicated by the coefficients of determination R^2^ on the test set equal to 0.78 and 0.71, respectively (File S4 at https://doi.org/10.5061/dryad.x95x69pj5). Moreover, the degree of similarity between actual and predicted phenotype was higher in the competition *vs* BL225C compared to those of the competition *vs* AK83, suggesting that the generated model well predicted the competition phenotype in this dataset (File S5C at https://doi.org/10.5061/dryad.x95x69pj5). In terms of model-evaluation metrics, the linear regression model was unsuccessful in defining the competition against Rm1021 for the test set (File S4 at https://doi.org/10.5061/dryad.x95x69pj5), as suggested by the negative value of averaged R^2^ obtained (equal to -0.18, File S4 at https://doi.org/10.5061/dryad.x95x69pj5). Precisely, the negative value indicates that the mean of the phenotype values of training samples have more predictive power on the test set than the model itself (for details see the r2_score function foreseen in the scikit-learn package and reported on [https://scikit-learn.org/stable/modules/model_evaluation.html#r2-score]). As also showed by the comparison between predicted and actual phenotype, the predictive capacity of the model was greatly inaccurate for those strains that displayed low or medium-low competition capabilities (*S. meliloti* T073, USDA1157, M270, Rm41, and HM006) (File S5A at https://doi.org/10.5061/dryad.x95x69pj5), in a competition where most of the strains showed medium-high capacities (Fig. 1A).

Considering single plus mixed nodule occupancy, the linear regression model of the BL225C competition showed an increase of the prediction power on the test set (R^2^ of test set equal to 0.87, File S4 at https://doi.org/10.5061/dryad.x95x69pj5), as also confirmed by the high similarity between actual and predicted phenotype (File S5E at https://doi.org/10.5061/dryad.x95x69pj5). Differently, the performance of the linear regression model in the competition versus AK83 significantly changed compared to the previous analyses (File S4 at https://doi.org/10.5061/dryad.x95x69pj5). Indeed, the prediction was unreliable/worthless/inconsistent in defining the competition, as hinted by the negative averaged R^2^ value in the test sets (File S4 at https://doi.org/10.5061/dryad.x95x69pj5). Accordingly, the model showed a clear lacking predictive ability, calculating/assigning the same value of predicted phenotype for all strains except for Rm41, T073, and 2011 (File S5E at https://doi.org/10.5061/dryad.x95x69pj5). Alike the previous results, the linear regression model failed in predicting the competition against Rm1021, as hinted by the negative averaged R^2^ for the test (File S4 at https://doi.org/10.5061/dryad.x95x69pj5) and by the inaccuracy/disagreement of predicted phenotypes compared to actual phenotypes (File S5D at https://doi.org/10.5061/dryad.x95x69pj5).

Overall, these results suggest that the trained linear regression models can well predict the most contrasting competition phenotype detected in the competition versus BL225C, evaluated as both single nodules occupancy and single plus mixed nodules occupancy.

**Supplementary methods**

**Nodulation and acetylene reduction assays***. Medicago sativa* (cv. Maravigliosa) seedlings were surface sterilized with 70% ethanol for 1 min, rinsed with sterile ddH_2_O, treated with 2.5% sodium hypochlorite for 5 min, and washed 20 times with sterile ddH_2_O. Sterilized seeds were then let germinate on the cover of sterile plastic Petri dishes upside down for 4 days in the dark at room temperature. Seedlings were transferred in plastic pots containing a sterilized mixture of sand and vermiculite (ratio 2:3) and supplied with 120 ml of sterilized Nitrogen-free solution (1mM CaCl_2_ 2H_2_0, 0.1 mM KCl, 0.8 mM MgSO_4_ 7H_2_O, 10 µM Fe EDTA, 35 µM H_3_BO_3_, 9 µM MnCl_2_ 4H_2_O, 0.8 µM ZnCl_2_, 0.5 µM Na_2_MoO_4_ 2H_2_O, 0.3 µM CuSO_4_ 5H_2_O, 3.68 mM KH_2_PO_4_, 4 mM Na_2_HPO_4_ pH=6.5) (1). Seedlings were grown for 3 additional days before inoculation with *S. meliloti* strains. The strains were grown at 30 °C to late exponential phase (OD_600_ < 0.6 - 0.8), washed 2 times in Nitrogen-free solution, and then adjusted to an OD_600_ = 0.05 in Nitrogen-free solution. Nine plants for strains were inoculated with aliquots of 500 µl of cell suspension of 5×10^7^ CFU/ml and grown in a growth chamber maintained at 23 °C with a 16-h photoperiod. The same amount of Nitrogen-free solution was added to negative control plants (C-). After 28 days, the epicotile length, number of nodules, and dry weight were measured.

For the acetylene-reduction assay, *M. sativa* plants were grown as described above. After 28 days, plants were collected in 100 ml glass flasks (3 plants/flask) and sealed with gas-thigh silicone caps. Aliquots of 10 ml of acetylene were injected into the flasks and, after 40 min, the ethylene concentration was measured by using a 7890B gas chromatograph system (Agilent technologies; California, USA), equipped with a 5975 Mass selective detector. Chromatographic analyses were performed in the following conditions: initial temperature, 40°C (isocratic for 10 min), gas flow (helium) 4 ml/min, injection 500 µl (gas syringe) at a split ratio of 5:1. Nitrogen fixation rates were expressed in nanomoles of produced ethylene *per* hour, *per* plant.

**Annotation and phylogenetic analyses.** *S. meliloti* genomes were retrieved from the NCBI Genome Database (GenBank codes are reported in Table S1). Genome annotation of 13 *S. meliloti* strains was completed using Prokka (version 1.13) bacterial genome annotation tool (2). Genome annotations are available as File S6 (at https://doi.org/10.5061/dryad.x95x69pj5). The pangenome of the 13 *S. meliloti* strains was constructed with Roary 3.11.3 (3) using default settings to construct a whole-genome phylogeny. Core genes alignment, obtained with Roary, was used to infer the evolutionary relationship of the strains tested as competitors. The evolutionary distances were computed using the Maximum Composite Likelihood method and are in the units of the number of base substitutions per site (4). The evolutionary history was reconstructed using the UPGMA method (bootstrap test of 1000 replicates). All ambiguous positions were removed for each sequence pair (pairwise deletion option). All evolutionary analyses were conducted using MEGA X software (5).

**Mapping procedure.** For each competing strain tested, the genome position of *k*-mers associated with the phenotype (competition against BL225C strain) was detected using the R package Biostrings (version 2.54) (6). Only *k*-mers aligning without mismatches or gaps on the positive or negative strand of the reference were taken into account to reflect the pipeline used by PhenotypeSeeker, which does not allow for mismatches. Absolute positions of *k*-mers were then transformed into relative ones based on genomic annotations following four rules:

1. If a *k*-mer was mapped inside a gene, its position was set to 0 independently from the strand
2. If a *k*-mer was mapped outside a gene on the positive strand, its position was adjusted by subtracting the starting position of the nearest gene. Since the starting position of the nearest gene on the plus strand is always greater than the starting position of the *k*-mer, the relative position will always be a negative value representing the number of bases ahead of the sequence of the gene on the reference genome.
3. If a *k*-mer was mapped outside a gene on the negative strand, its position was calculated by subtracting the starting position of the *k*-mer to the ending position of the gene. Analogously to the previous calculation, the starting position of the *k*-mer will always be greater than the ending position of the gene on the minus strand, thus the relative position of the *k*-mer will always be a negative value representing the number of bases behind the sequence of the gene on the reference genome.
4. If a *k*-mer was mapped ahead of a gene on the positive strand and behind a gene on the negative strand (namely “between” two genes oriented in different directions), its position was calculated as reported in 1 and 2. Since both relative positions may be valid they were both reported and considered in downstream analyses. Relative positions obtained were then used to extract the predicted protein-coding sequences (CDS) and regulatory regions mapped by 51 *k*-mers (with a *P* value = 1.31 10^-04^) by selecting those with a relative position equal to 0 and higher than -600 respectively.

**Supplemental text references**

1. Poole PS, Blyth A, Reid CJ, Walters K. 1994. myo-Inositol catabolism and catabolite regulation in *Rhizobium leguminosarum* bv. *viciae*. Microbiology 140:2787–2795.

2. Seemann T. 2014. Prokka: Rapid prokaryotic genome annotation. Bioinformatics 30:2068–2069.

3. Page AJ, Cummins CA, Hunt M, Wong VK, Reuter S, Holden MTG, Fookes M, Falush D, Keane JA, Parkhill J. 2015. Roary: Rapid large-scale prokaryote pan genome analysis. Bioinformatics 31:3691–3693.

4. Tamura K, Nei M, Kumar S. 2004. Prospects for inferring very large phylogenies by using the neighbor-joining method. Proc Natl Acad Sci U S A 101:11030–11035.

5. Kumar S, Stecher G, Li M, Knyaz C, Tamura K. 2018. MEGA X: Molecular evolutionary genetics analysis across computing platforms. Mol Biol Evol 35:1547–1549.

6. Pagès H, Aboyoun P, Gentleman R, DebRoy S. 2017. Biostrings: Efficient manipulation of biological strings. R Packag version 2460.

7. Galardini M, Mengoni A, Brilli M, Pini F, Fioravanti A, Lucas S, Lapidus A, Cheng J, Goodwin L, Pitluck S, Land M, Hauser L, Woike T, Mikhailova N, Ivanova N, Daligault H, Bruce D, Detter C, Tapia R, Han C, Teshima H, Mocali S, Bazzicalupo M, Biondi EG. 2011. Exploring the symbiotic pangenome of the nitrogen-fixing bacterium *Sinorhizobium meliloti*. BMC Genomics 12:235.

8. Giuntini E, Mengoni A, De Filippo C, Cavalieri D, Aubin-Horth N, Landry CR, Becker A, Bazzicalupo M. 2005. Large-scale genetic variation of the symbiosis-required megaplasmid pSymA revealed by comparative genomic analysis of *Sinorhizobium meliloti* natural strains. BMC Genomics 6:158.

9. Galibert F, Finan TM, Long SR, Puhler A, Abola P, Ampe F, Barloy-Hubler F, Barnett MJ, Becker A, Boistard P, Bothe G, Boutry M, Bowser L, Buhrmester J, Cadieu E, Capela D, Chain P, Cowie A, Davis RW, Dreano S, Federspiel NA, Fisher RF, Gloux S, Godrie T, Goffeau A, Golding B, Gouzy J, Gurjal M, Hernandez-Lucas I, Hong A, Huizar L, Hyman RW, Jones T, Kahn D, Kahn ML, Kalman S, Keating DH, Kiss E, Komp C, Lelaure V, Masuy D, Palm C, Peck MC, Pohl TM, Portetelle D, Purnelle B, Ramsperger U, Surzycki R, Thebault P, Vandenbol M, Vorholter FJ, Weidner S, Wells DH, Wong K, Yeh KC, Batut J. 2001. The composite genome of the legume symbiont *Sinorhizobium meliloti*. Science 293:668–672.

10. Meade HM, Long SR, Ruvkun GB, Brown SE, Ausubel FM. 1982. Physical and genetic characterization of symbiotic and auxotrophic mutants of *Rhizobium meliloti* induced by transposon Tn5 mutagenesis. J Bacteriol 149:114–122.

11. Carelli M, Gnocchi S, Fancelli S, Mengoni A, Paffetti D, Scotti C, Bazzicalupo M. 2000. Genetic diversity and dynamics of *Sinorhizobium meliloti* populations nodulating different alfalfa cultivars in Italian soils. Appl Environ Microbiol 66:4785–4789.

12. Sugawara M, Epstein B, Badgley BD, Unno T, Xu L, Reese J, Gyaneshwar P, Denny R, Mudge J, Bharti AK, Farmer AD, May GD, Woodward JE, Medigue C, Vallenet D, Lajus A, Rouy Z, Martinez-Vaz B, Tiffin P, Young ND, Sadowsky MJ. 2013. Comparative genomics of the core and accessory genomes of 48 *Sinorhizobium* strains comprising five genospecies. Genome Biol 14:R17.

13. Nelson M, Guhlin J, Epstein B, Tiffin P, Sadowsky MJ. 2018. The complete replicons of 16 *Ensifer meliloti* strains offer insights into intra- and inter-replicon gene transfer, transposon-associated loci, and repeat elements. Microb Genom 4:e000174.

14. Nagymihály M, Vásarhelyi BM, Barrière Q, Chong T-M, Bálint B, Bihari P, Hong K-W, Horváth B, Ibijbijen J, Amar M, Farkas A, Kondorosi É, Chan K-G, Gruber V, Ratet P, Mergaert P, Kereszt A. 2017. The complete genome sequence of *Ensifer meliloti* strain CCMM B554 (FSM-MA), a highly effective nitrogen-fixing microsymbiont of *Medicago truncatula Gaertn*. Stand Genomic Sci 12:75.

15. Kazmierczak T, Nagymihály M, Lamouche F, Barrière Q, Guefrachi I, Alunni B, Ouadghiri M, Ibijbijen J, Kondorosi É, Mergaert P, Gruber V. 2017. Specific host-responsive associations between *Medicago truncatula* accessions and *Sinorhizobium* strains. Mol Plant Microbe Interact 30:399–409.

16. Weidner S, Baumgarth B, Göttfert M, Jaenicke S, Pühler A, Schneiker-Bekel S, Serrania J, Szczepanowski R, Becker A. 2013. Genome Sequence of *Sinorhizobium meliloti* Rm41. Genome Announc 1:e00013-12.

17. Szende K, Ördögh F. 1960. Die Lysogenie von *Rhizobium meliloti*. Naturwissenschaften 47:404–405.

18. Martinez-Abarca F, Martinez-Rodriguez L, Lopez-Contreras JA, Jimenez-Zurdo JI, Toro N. 2013. Complete genome sequence of the alfalfa symbiont *Sinorhizobium/Ensifer meliloti* strain GR4. Genome Announc 1:e00174-12.

19. Sallet E, Roux B, Sauviac L, Jardinaud MF, Carrere S, Faraut T, de Carvalho-Niebel F, Gouzy J, Gamas P, Capela D, Bruand C, Schiex T. 2013. Next-generation annotation of prokaryotic genomes with EuGene-P: application to *Sinorhizobium meliloti* 2011. DNA Res 20:339–354.

20. Schneiker-Bekel S, Wibberg D, Bekel T, Blom J, Linke B, Neuweger H, Stiens M, Vorholter FJ, Weidner S, Goesmann A, Puhler A, Schluter A. 2011. The complete genome sequence of the dominant *Sinorhizobium meliloti* field isolate SM11 extends the *S. meliloti* pan-genome. J Biotechnol 155:20–33.

21. Stiens M, Schneiker S, Keller M, Kuhn S, Pühler A, Schlüter A. 2006. Sequence analysis of the 144-kilobase accessory plasmid pSmeSM11a, isolated from a dominant *Sinorhizobium meliloti* strain identified during a long-term field release experiment. Appl Environ Microbiol 72:3662–3672.

22. Wibberg D, Blom J, Ruckert C, Winkler A, Albersmeier A, Puhler A, Schluter A, Scharf BE. 2013. Draft genome sequence of *Sinorhizobium meliloti* RU11/001, a model organism for flagellum structure, motility and chemotaxis. J Biotechnol 168:731–733.

23. Krupski G, Götz R, Ober K, Pleier E, Schmitt R. 1985. Structure of complex flagellar filaments in *Rhizobium meliloti*. J Bacteriol 162:361–366.

24. Galardini M, Bazzicalupo M, Biondi E, Brambilla E, Brilli M. 2013. Permanent draft genome sequences of the symbiotic nitrogen fixing *Ensifer meliloti* strains BO21CC and AK58. Stand Genomic Sci 9:325–333.

25. Checcucci A, Azzarello E, Bazzicalupo M, Galardini M, Lagomarsino A, Mancuso S, Marti L, Marzano MC, Mocali S, Squartini A, Zanardo M, Mengoni A. 2016. Mixed nodule infection in *Sinorhizobium meliloti–Medicago sativa* symbiosis suggest the presence of cheating behavior. Front Plant Sci 7:835.

26. Smit P, Raedts J, Portyanko V, Debellé F, Gough C, Bisseling T, Geurts R. 2005. NSP1 of the GRAS protein family is essential for rhizobial Nod factor-induced transcription. Science (80- ) 308:1789–1791.

27. Cheng HP, Walker GC. 1998. Succinoglycan is required for initiation and elongation of infection threads during nodulation of alfalfa by *Rhizobium meliloti*. J Bacteriol 180:5183–5191.
