# Supplementary material for: Competitiveness for Nodule Colonization in Sinorhizobium meliloti: Combined In Vitro-Tagged Strain Competition and Genome-Wide Association Analysis
Source: mSystems. 2021 Jul 27;6(4):e00550-21. doi: 10.1128/mSystems.00550-21 (PMC8407117; doi:10.1128/mSystems.00550-21)
Supplement: TABLE S3 [file msystems.00550-21-st003.docx]

**Table S3**. Strains and plasmids used in this work.

| **Species** | **Strains (or plasmids)** | **Source/Description** | **Resistances** | **Reference** |
| --- | --- | --- | --- | --- |
| *Sinorhizobium meliloti* | AK83 |  |  | (8) |
|  | 1021 | SU47 *str*-21 | Str^1^ | (10) |
|  | BL225C |  |  | (11) |
|  | KH46 |  |  | (12) |
|  | CCMM B554 |  |  | (15) |
|  | T073 |  |  | (12) |
|  | Rm41 |  |  | (17) |
|  | HM006 |  |  | (12) |
|  | GR4 |  |  | (18) |
|  | 2011 | SU47 | Str | - |
|  | USDA1157 |  |  | (13) |
|  | KH35c |  |  | (12) |
|  | SM11 |  |  | (21) |
|  | RU11/001 |  | Str | (23) |
|  | M270 |  |  | (12) |
|  | AK58 |  |  | (8) |
|  | BM685 | AK83 pBHR - mRFP. | Rif^2^ & Tc^3^ | (25) |
|  | BM687 | 1021 pBHR - mRFP. | Str & Tc | (25) |
|  | GE0346 | BL225C pBHR – mRFP. | Rif & Tc | This work |
|  | GE0323 | KH46 pHC60 | Rif & Tc | This work |
|  | GE0326 | CCMM B554 pHC60 | Rif & Tc | This work |
|  | GE0327 | T073 pHC60 | Rif & Tc | This work |
|  | GE0328 | Rm41 pHC60 | Rif & Tc | This work |
|  | GE0329 | HM006 pHC60 | Rif & Tc | This work |
|  | GE0330 | GR4 pHC60 | Rif & Tc | This work |
|  | GE0339 | 2011 pHC60 | Str & Tc | This work |
|  | GE0341 | USDA1157 pHC60 | Rif & Tc | This work |
|  | GE0342 | KH35c pHC60 | Rif & Tc | This work |
|  | GE0345 | SM11 pHC60 | Rif & Tc | This work |
|  | GE0357 | RU11/001 pHC60 | Rif & Tc | This work |
|  | GE0359 | M270 pHC60 | Rif & Tc | This work |
|  | GE0360 | AK58 pHC60 | Rif & Tc | This work |
| *Escherichia coli* | BM266 | S17-1 λpir pHC60 | Tc | (25) |
|  | BM679 | S17-1 λpir pBHR- mRFP | Tc | (25) |
| Plasmids | pBHR - mRFP | Constitutive expression of RFP | Tc | (26) |
|  | pHC60 | Constitutive expression of GFP | Tc | (27) |
